# Close relatives of MERS-CoV in bats use ACE2 as their functional receptors

**DOI:** 10.1038/s41586-022-05513-3

**Published:** 2022-12-07

**Authors:** Qing Xiong, Lei Cao, Chengbao Ma, M. Alejandra Tortorici, Chen Liu, Junyu Si, Peng Liu, Mengxue Gu, Alexandra C. Walls, Chunli Wang, Lulu Shi, Fei Tong, Meiling Huang, Jing Li, Chufeng Zhao, Chao Shen, Yu Chen, Huabin Zhao, Ke Lan, Davide Corti, David Veesler, Xiangxi Wang, Huan Yan

**Affiliations:** 1 State Key Laboratory of Virology, Institute for Vaccine Research and Modern Virology Research Center, College of Life Sciences, TaiKang Center for Life and Medical Sciences, Wuhan University, Wuhan, China; 2 CAS Key Laboratory of Infection and Immunity, National Laboratory of Macromolecules, Institute of Biophysics, Chinese Academy of Sciences, Beijing, China; 3 Department of Biochemistry, University of Washington, Seattle, WA USA; 4 Howard Hughes Medical Institute, Seattle, WA USA; 5 Department of Ecology, Tibetan Centre for Ecology and Conservation at WHU-TU, Hubei Key Laboratory of Cell Homeostasis, College of Life Sciences, Wuhan University, Wuhan, China; 6 Humabs BioMed SA, subsidiary of Vir Biotechnology, Bellinzona, Switzerland; 7 University of Chinese Academy of Sciences, Beijing, China

**Keywords:** Virus-host interactions, Cryoelectron microscopy, Viral evolution

## Abstract

Middle East respiratory syndrome coronavirus (MERS-CoV) and several bat coronaviruses use dipeptidyl peptidase-4 (DPP4) as an entry receptor^1–4^. However, the receptor for NeoCoV—the closest known MERS-CoV relative found in bats—remains unclear^5^. Here, using a pseudotype virus entry assay, we found that NeoCoV and its close relative, PDF-2180, can efficiently bind to and use specific bat angiotensin-converting enzyme 2 (ACE2) orthologues and, less favourably, human ACE2 as entry receptors through their receptor-binding domains (RBDs) on the spike (S) proteins. Cryo-electron microscopy analysis revealed an RBD–ACE2 binding interface involving protein–glycan interactions, distinct from those of other known ACE2-using coronaviruses. We identified residues 337–342 of human ACE2 as a molecular determinant restricting NeoCoV entry, whereas a NeoCoV S pseudotyped virus containing a T510F RBD mutation efficiently entered cells expressing human ACE2. Although polyclonal SARS-CoV-2 antibodies or MERS-CoV RBD-specific nanobodies did not cross-neutralize NeoCoV or PDF-2180, an ACE2-specific antibody and two broadly neutralizing betacoronavirus antibodies efficiently inhibited these two pseudotyped viruses. We describe MERS-CoV-related viruses that use ACE2 as an entry receptor, underscoring a promiscuity of receptor use and a potential zoonotic threat.

## Abstract (web summary)

NeoCoV and its close relative, PDF-2180, can efficiently bind to and use specific bat ACE2 orthologues and, less favourably, human ACE2 as entry receptors through their receptor-binding domains on the spike protein.

## Main

Coronaviruses are a large family of enveloped positive-strand RNA viruses classified into four genera: alpha-, beta-, gamma- and deltacoronaviruses. Alpha- and betacoronaviruses typically infect mammals such as bats and humans, whereas gamma- and deltacoronaviruses mainly infect birds and occasionally mammals^6,7^. It is thought that the origins of most coronaviruses that infect humans can be traced back to their close relatives in bats—the most important animal reservoir of mammalian coronaviruses^8^. Cross-species transmission of coronaviruses has led to three major outbreaks in the past two decades caused by SARS-CoV, MERS-CoV and, most recently, SARS-CoV-2^9–12^.

MERS-CoV belongs to lineage C of betacoronaviruses (merbecovirus subgenus) and has been reported to have a high case–fatality rate of approximately 35% (ref. ^13^). Merbecoviruses have also been found in several animal species, including camels, hedgehogs and bats. Although camels are confirmed intermediate hosts for MERS-CoV, bats, especially species in the family of Vespertilionidae, are widely considered to be the evolutionary source of MERS-CoV or its immediate ancestor^14^.

Specific coronavirus recognition of host receptors is usually mediated by the RBD (or domain B) within the spike protein S_1_ subunit^15,16^. Three out of four well-characterized human coronavirus receptors are transmembrane proteases, namely ACE2, DPP4 and aminopeptidase N (APN)^1,17,18^. By contrast, carcinoembryonic antigen-related cell adhesion molecule 1 (CEACAM1) interacts with the murine hepatitis virus spike N-terminal domain (domain A)^19,20^. Notably, the same receptor can be shared by distantly related coronaviruses with structurally distinct RBDs. For example, NL63 (an alphacoronavirus) uses ACE2 as an entry receptor, which is also widely used by sarbecoviruses (betacoronavirus lineage B)^21^. APN is a second example of cross-genera receptor use, as it mediates the entry of several alphacoronaviruses and a deltacoronavirus (PDCoV)^7^. However, DPP4 is known to be an entry receptor only for some merbecoviruses (betacoronavirus lineage C), such as MERS-CoV, HKU4, HKU25 and related strains^2–4,22^.

Several merbecoviruses do not use DPP4 for entry, and their receptor use remains unclear, including the bat coronaviruses NeoCoV, PDF-2180, HKU5 and hedgehog coronaviruses EriCoV-HKU31^5,23–25^. NeoCoV is the closest relative of MERS-CoV (85% nucleotide sequence identity at the whole-genome level) and was identified in *Neoromicia capensis* (Cape serotine, or *Laephotis capensis*, formerly classified in *Neoromicia* before phylogenetic analysis found that it belongs to *Laephotis*). NeoCoV was previously erroneously assumed to be sampled from *Neoromicia zuluensis* bats in South Africa^26,27^. PDF-2180, which is closely related to NeoCoV phylogenetically (around 91% amino acid sequence identity in the S_1_ subunits), infects *Pipisrellus hesperidus* bats, which are native to Southwest Uganda^23,28^. The marked sequence divergence of the NeoCoV and PDF-2180 S_1_ subunits relative to MERS-CoV (around 44–45% amino acid sequence identity) probably explains why these viruses do not use DPP4 as an entry receptor^23^.

Here we report that NeoCoV and PDF-2180 use bat ACE2 as an entry receptor. We determined the cryo-electron microscopy (cryo-EM) structures of the PDF-2180 spike trimer as well as of the NeoCoV and PDF-2180 RBDs bound to *Pipistrellus pipistrellus* ACE2, revealing a binding mode that is distinct from that observed for SARS-CoV-2 and NL63. Although NeoCoV and PDF-2180 use human ACE2 (hACE2) suboptimally, adaptive mutations of this group of viruses might result in future spillover events.

## Evidence of ACE2 use

**Fig. 1:**
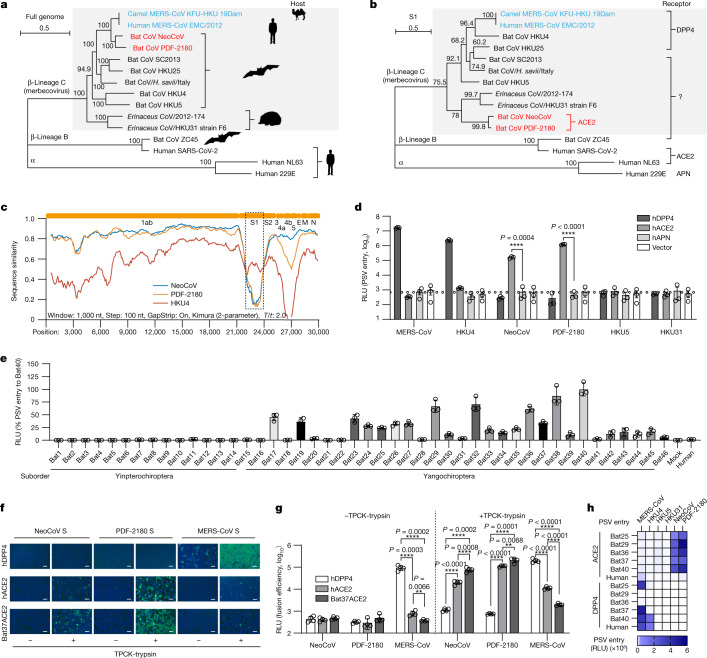
NeoCoV and PDF-2180 use ACE2 but not DPP4 for efficient entry. **a**,**b**, Phylogenetic analysis of merbecoviruses (grey) based on whole-genome nucleotide sequences (**a**) and S_1_ amino acid sequences (**b**). NL63 and 229E were set as outgroups. Host and receptor use are indicated. For the scale bars, 0.5 represents two nucleotide substitutions per site. **c**, Simplot analysis showing the whole-genome nucleotide sequence similarity of three merbecoviruses compared with MERS-CoV. The boundaries of regions encoding MERS-CoV proteins are indicated at the top. The box delineated by a dashed line underscores the divergence of the S_1_ subunit. *T*/*t*, transition/transversion ratio. **d**, Pseudotyped S virus entry efficiency of six merbecoviruses in BHK-21 cells transiently expressing hACE2, hDPP4 or hAPN. The dashed lines indicate the baseline of background signals (mean values of vector-only groups). Data are mean ± s.e.m., representative of three independent experiments. *n* = 3 biologically replicates. Statistical analysis was performed using two-tailed unpaired Student’s *t*-tests. PSV, pseudotyped virus. **e**, The entry efficiency of NeoCoV S pseudotyped virus in HEK293T stable cell lines expressing different bat ACE2 orthologues as indicated by luciferase activity. Data are mean ± s.d. of biological triplicates examined over three independent infection assays. **f**,**g**, NeoCoV, PDF-2180 and MERS-CoV S-mediated cell–cell fusion analysis based on DSP assays in HEK293T cells stably expressing the indicated receptors. TPCK-trypsin, TPCK-treated trypsin treatment (10 μg ml^−1^). eGFP intensity (**f**) and live-cell *Renilla* luciferase activity (**g**) are shown. Data are mean ± s.d. of *n* = 4 biologically independent cells. Three independent cell–cell fusion assays are shown. Statistical analysis was performed using two-tailed unpaired Student’s *t*-tests. For **f**, scale bars, 200 μm. **h**, Pseudotyped virus entry efficiency of six merbecoviruses in HEK293T cells stably expressing the indicated bat ACE2 or DPP4 orthologues. Data are representative results of three independent experiments and plotted by the mean of the biological triplicate. RLU, relative light units. **P < 0.01, ****P < 0.001. Source data

**Extended Data Fig. 1:**
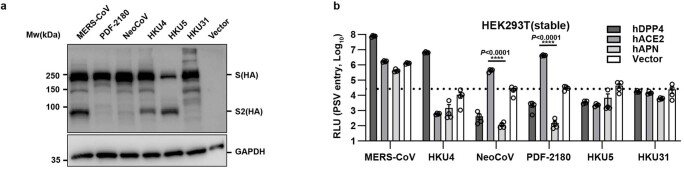
Pseudotyped virus entry efficiency of six merbecoviruses in HEK293T cells stably expressing the indicated human ACE2, DPP4, or APN. **a**, The expression levels of coronaviruses S proteins in transiently transfected HEK293T cells were analysed by Western blot detecting the C-terminal fused HA tags. GAPDH was used as a loading control. Experiments were performed in triplicates. **b**, Pseudotyped S virus entry efficiency of six merbecoviruses in HEK293T cells stably expressing hACE2, hDPP4, or hAPN. The dashed lines indicate the baseline of background signals (mean values of vector-only groups with MERS-CoV excluded). Blot representative of experiments of three independent transfections for generating each pseudoviruses. Data are presented as mean ± SEM (n = 4 biologically independent infected cells), representative of three independent infection assays with similar results. Two-tailed unpaired Student’s *t*-test; ****P < 0.001. RLU: relative light units. Source data

To shed light on the evolutionary relationship among merbecoviruses, especially NeoCoV and PDF-2180, we conducted a phylogenetic analysis of the sequences of several human and animal coronaviruses. Maximum-likelihood phylogenetic reconstructions based on complete genome nucleotide sequences showed that NeoCoV and PDF-2180 form a sister clade to MERS-CoV (Fig. 1a). By contrast, a phylogenetic tree based on amino acid sequences of the S_1_ subunit demonstrated that NeoCoV and PDF-2180 showed a distant evolutionary relationship with MERS-CoV but are closely related to hedgehog coronaviruses (EriCoVs) (Fig. 1b). A similarity plot analysis (Simplot) highlights that the S_1_ subunits of NeoCoV and PDF-2180 are even more divergent from MERS-CoV than is the case for HKU4 (Fig. 1c). We first tested whether human DPP4 (hDPP4) could support the cell entry of several merbecoviruses using a pseudotype virus assay (Extended Data Fig. 1a). The result revealed that MERS-CoV S and HKU4 S pseudotyped viruses showed robust entry in BHK-21-hDPP4 and HEK293T-hDPP4 cells, although there is detectable MERS-CoV pseudovirus entry in other HEK293T cell lines, probably due to weak endogenous expression of hDPP4 (Fig. 1d and Extended Data Fig. 1b). Notably, we detected a significant increase in the entry of NeoCoV S and PDF-2180 S pseudotyped viruses in hACE2-expressing cells but not in hAPN-expressing cells, both of which were initially considered to be negative control cells (Fig. 1d and Extended Data Fig. 1b).

**Extended Data Fig. 2:**
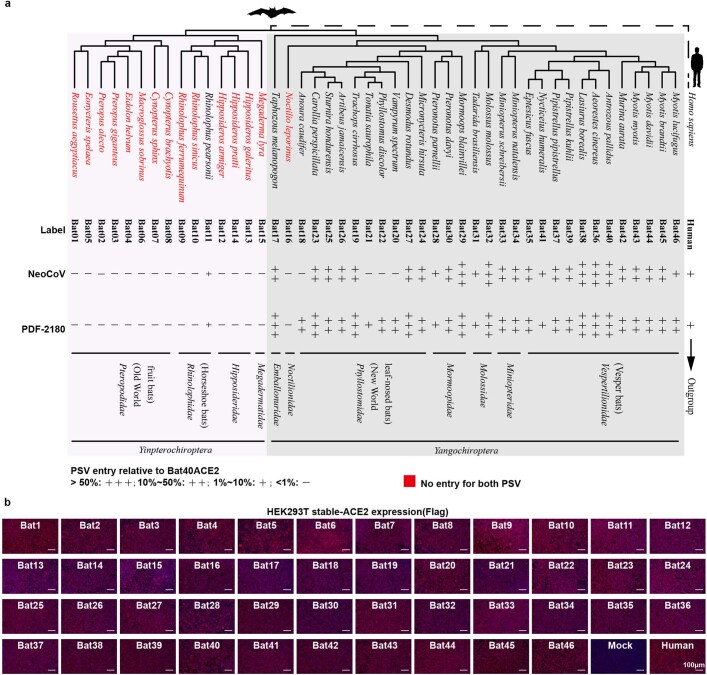
Bat ACE2 orthologs tested in this study and their ability to support the entry of NeoCoV and PDF-2180. **a**, Phylogenetic tree and assessment of the ability of a panel of ACE2 orthologs from 46 bat species to support NeoCoV and PDF-2180 S-mediated entry (relative to Bat40 ACE2). ACE2 orthologs deficient in supporting the entry of both viruses are highlighted in red. The GenBank accession numbers and protein sequences were summarized in Supplementary Table 4. **b**, Immunofluorescence assay detecting the 46 bat ACE2 orthologs C-terminal 3×Flag tag in the HEK293T stable cells. Mock indicates cells were transduced with lentiviruses produced by empty vector only. Data representative of three independent immunostaining assays with similar results.

**Extended Data Fig. 3:**
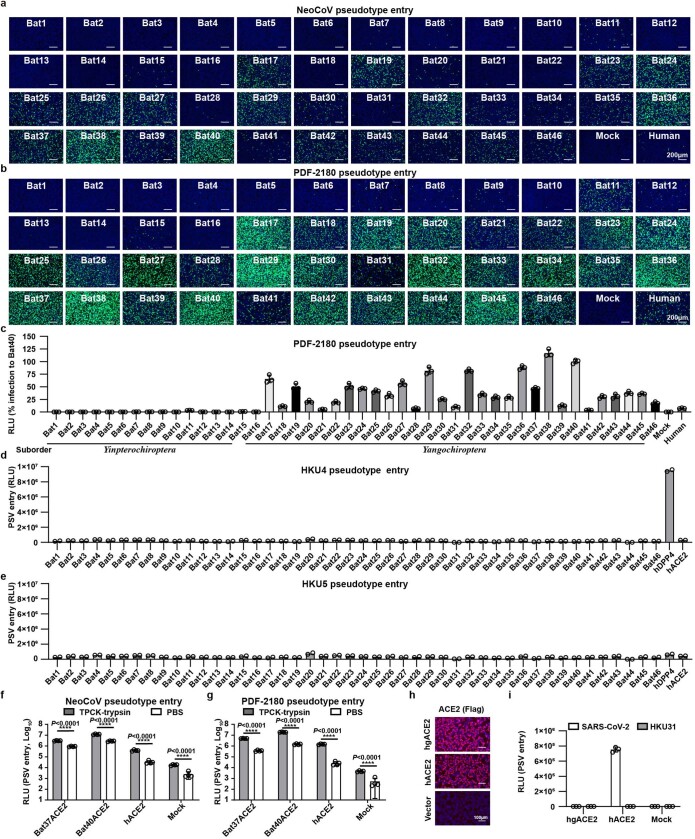
Entry of indicated pseudoviruses in HEK293T cells stably expressing different bat ACE2 orthologs. **a**-**e**, Entry efficiency of NeoCoV (**a**) PDF-2180 (**b**-**c**), HKU4 (**d**), and HKU5 (**e**) S pseudotyped viruses as indicated by GFP (**a**-**b**) or luciferase (**c**-**e)** intensity. Data representative of two independent infection assays. Data are presented as mean ± SD (biological triplicates for **a**-**c** and biological duplicates for **d**-**e**). **f**-**g**, TPCK-treated trypsin treatment (100 μg/ml) significantly enhanced NeoCoV (**f**) and PDF-2180 (**g**) S pseudotyped virus entry in HEK293T cells expressing different ACE2 orthologs. Data are presented as mean ± SD (biological triplicates of infected cells), representative of two independent experiments. Two-tailed unpaired Student’s *t*-test; ****P < 0.001. **h**, The expression level of ACE2 was evaluated by immunofluorescence detecting the C-terminal fused 3×Flag tag. Experiments performed twice with similar results and representative data are shown. **i**, Entry of SARS-CoV-2 and HKU31 S pseudotyped viruses into cells expressing hACE2 or hedgehog hgACE2. Representative data are presented as mean ± SD (biological triplicates of infected cells) examined over two independent experiments. Source data

**Extended Data Fig. 4:**
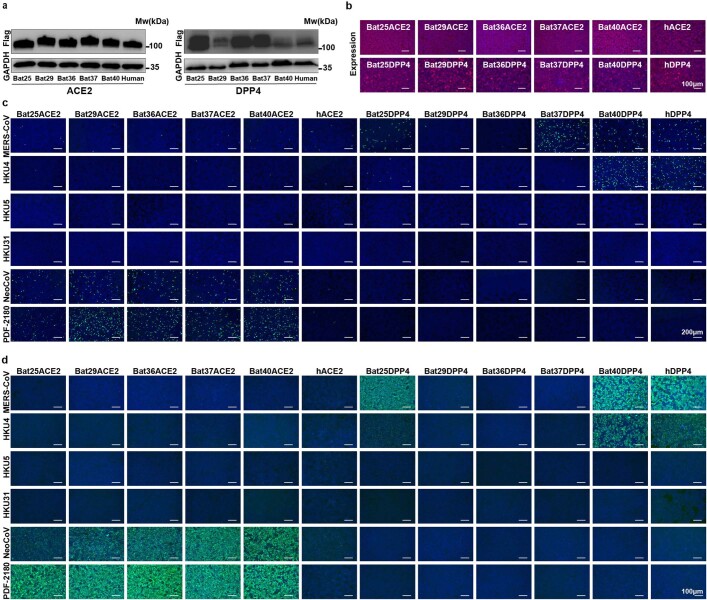
ACE2 and DPP4 receptor usage of different merbecoviruses. **a**, Western blot analysis of the expression levels of ACE2 and DPP4 orthologs in HEK293T cells. Blot representative of immunoblotting based on biological independent duplicates of transfected cells. **b**, Evaluation of bat ACE2 expression level by immunofluorescence assay detecting the C-terminal 3×Flag tag. Data representative of two independent immunofluorescence assays based on two different transfection experiments. **c**-**d**, Pseudotyped virus entry (**c**) and RBD binding (**d**) of different CoVs on HEK293T cells expressing different ACE2 or DPP4 orthologs. The experiment was repeated independently twice with similar results, and representative data are shown.

**Extended Data Fig. 5:**
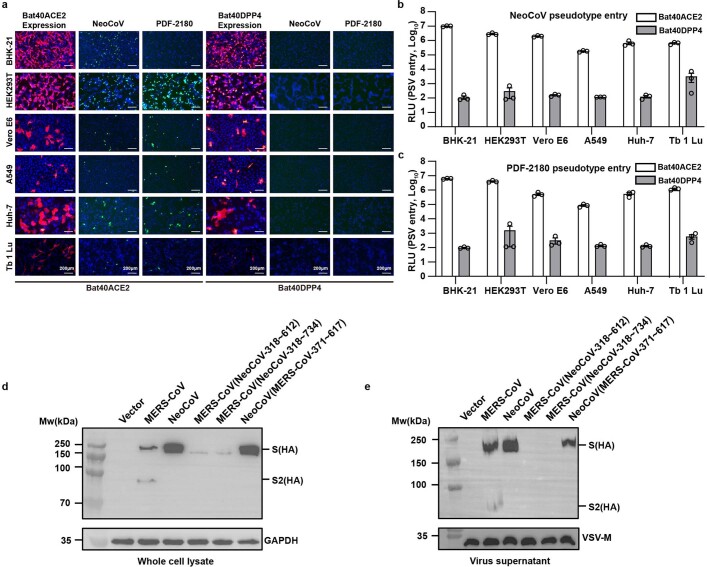
ACE2 and Domain B dependent entry of NeoCoV, PDF-2180 S pseudotyped viruses in different cell types. **a**-**c**, BHK-21, HEK293T, Vero E6, A549, Huh-7, and Tb 1 Lu cells were transfected with either Bat40ACE2 or Bat40DPP4. Expression, pseudotyped virus entry (GFP) (**a**), and luciferase intensity (**b**-**c**) were detected at 16 h post-infection. Data are presented as mean ± SD (biological triplicates). Data representative of two independent transfection and infection assays. **d**, Expression levels of the indicated chimeric viral S glycoproteins (anti-HA) in HEK293T whole cell lysates. GAPDH was used as a loading control. **e**, Chimeric viral S glycoproteins (anti-HA) and VSV-M level in pseudotyped viruses precipitated from virus-containing supernatant. Blots representative of two independent transfection assays for pseudovirus production. Source data

To investigate the possibility of more efficient use of bat ACE2 by these viruses, we screened an ACE2 library comprising 46 HEK293T stable cell lines individually expressing ACE2 orthologues spanning the Chiropteran phylogeny, as described in our previous study^29^ (Extended Data Fig. 2a,b and Supplementary Table 4). NeoCoV S and PDF-2180 S, but not HKU4 S or HKU5 S pseudotyped viruses, showed efficient entry in cells expressing ACE2 from most Yangochiropteran bats, whereas no or very limited entry was observed using cells expressing ACE2 from Yinpterochiropteran bats or humans (Fig. 1e and Extended Data Fig. 3a–e). Consistent with previous reports, NeoCoV and PDF-2180 S-mediated pseudotyped virus entry could be substantially enhanced by exogenous trypsin treatment^28^ (Extended Data Fig. 3f,g). As indicated by a dual split protein (DSP)-based fusion assay, ACE2 from Bat37 (Methods; Bat37ACE2) promoted more efficient cell–cell fusion than hACE2 in the presence of NeoCoV or PDF-2180 S, but not MERS-CoV S (Fig. 1f,g). The fact that human or hedgehog ACE2 did not support entry of EriCoV-HKU31 suggests that this virus may use a different receptor compared with NeoCoV and PDF-2180 (Extended Data Fig. 3h,i). Our results rule out bat DPP4 as a possible receptor for NeoCoV or PDF-2180, as none of the tested DPP4 orthologues could promote detectable entry of these two pseudotyped viruses (Fig. 1h and Extended Data Fig. 4a–d), in agreement with previous studies^23,28^. Pseudotyped virus entry assays were also conducted using several other cell types from various species, including a bat cell line Tb 1 Lu, ectopically expressing ACE2 or DPP4 from Bat40 (*Antrozous pallidus*), and each test yielded similar results (Extended Data Fig. 5a–c).

## Domain-B-mediated specific ACE2 binding

**Fig. 2:**
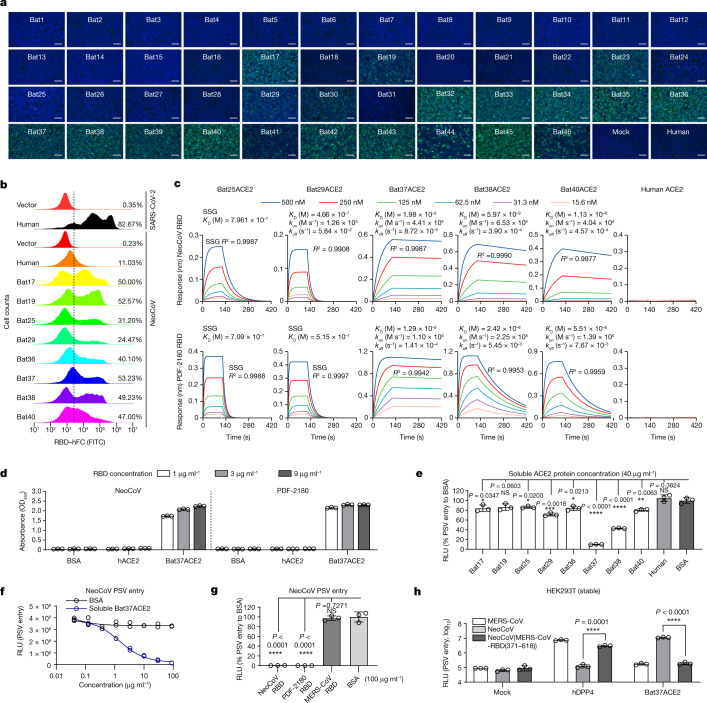
The domain B (RBD) of NeoCoV and PDF-2180 S proteins are required for species-specific ACE2 binding. **a**, Binding of NeoCoV RBD–hFc to bat ACE2 orthologues expressed on the surface of HEK293T cells analysed using a live-cell binding assay. Data are representative of three assays using independent preparations of proteins. Scale bars, 100 μm. **b**, Flow cytometry analysis of NeoCoV RBD–hFc binding to HEK293T cells expressing the indicated ACE2 orthologues. The ratio of positive cells compared with the vector control is indicated based on the threshold (dashed line). Data are mean values (*n* = 3 technical replicates), representative of three independent experiments. **c**, BLI assays analysing binding kinetics between NeoCoV RBD–hFc and PDF-2180-RBD–hFc with selected ACE2 ectodomains. The reported *K*_D,app_ values correspond to avidities due to the use of dimeric ACE2 constructs. SSG, steady-state affinity determination. Unfitted curves are shown in Supplementary Fig. 4a. **d**, ELISA assay showing the binding efficiency of NeoCoV and PDF-2180 RBD–hFc proteins to soluble ACE2 ectodomain proteins. Data are representative of two assays using independent preparations of proteins. Data are mean ± s.d. (technical triplicates). OD_450_, optical density at 450 nm. **e**, The inhibitory activity of soluble ACE2 against NeoCoV S pseudotyped virus entry in HEK293T-Bat37ACE2 cells. Data are mean ± s.d. *n* = 3 biologically independent cells. **f**, Concentration-dependent inhibition of NeoCoV S-mediated entry by soluble Bat37ACE2 in HEK293T-Bat37ACE2 cells. Data points represent biological duplicates. **g**, Evaluation of the inhibitory effect of NeoCoV, PDF-2180 or MERS-CoV RBD–hFc proteins on NeoCoV S pseudotyped virus entry in HEK293T-Bat37ACE2 cells. Data are mean ± s.d. (biologically triplicates). **h**, Entry of the MERS-CoV S, NeoCoV S or NeoCoV S chimera containing the MERS-CoV RBD (residues 371–618) pseudotyped viruses into HEK293T cells stably expressing one of the indicated receptors. Data are mean ± s.d. *n* = 3 independently infected cells. For **e**–**h**, data are representative of two independent infection assays. Statistical analysis was performed using two-tailed unpaired Student’s *t*-tests (**e**, **g** and **h**); *P < 0.05, ***P < 0.005; NS, not significant. Source data

The inability of NeoCoV and PDF-2180 to use DPP4 is consistent with their highly divergent domain B (putative RBD) sequence compared with MERS-CoV and HKU4. We produced RBD–hFc (domain B fused to human IgG Fc domain) proteins to verify whether this domain was responsible for ACE2 receptor engagement. Live-cell binding assays using NeoCoV RBD–hFc showed species-specific recognition of various cell-surface-expressed bat ACE2 orthologues, in agreement with the results of the pseudotyped virus entry assays (Fig. 2a). Specific binding of NeoCoV RBD–hFc to several representative bat ACE2 orthologues was also confirmed by flow cytometry (Fig. 2b). Using biolayer interferometry (BLI), we found that the NeoCoV and PDF-2180 RBDs bound with the highest avidity to *Pipistrellus pipistrellus* (Bat37) ACE2 (apparent binding affinity (*K*_D,app_) = 1.98 nM for NeoCoV and *K*_D,app_ = 1.29 nM for PDF-2180), whereas hACE2 binding was below the detection limit (Fig. 2c). An enzyme-linked immunosorbent assay (ELISA) also demonstrated the strong binding between NeoCoV and PDF-2180 RBDs and Bat37ACE2, but not hACE2 (Fig. 2d). As the ACE2 sequences of the hosts of NeoCoV and PDF-2180 are unknown, Bat37 represents the closest relative of the host of PDF-2180 (*P. hesperidus*) in our study. Although Bat37ACE2 is not the best receptor for supporting pseudotype entry, probably due to its moderate association rate (*k*_on_) with the RBDs of the two viruses, the observed dissociation rate (*k*_off_) is very slow, rendering it ideal for virus neutralization and cryo-EM analysis. The estimated binding affinities were further verified by competitive neutralization assays using soluble ACE2-ectodomain or RBD–hFc proteins. The entry of both NeoCoV and PDF-2180 S pseudotyped viruses was inhibited most efficiently by soluble Bat37ACE2 in a concentration-dependent manner (Fig. 2e,f). Moreover, the NeoCoV RBD–hFc and the PDF-2180 RBD–hFc neutralized NeoCoV S pseudotyped virus entry into cells expressing Bat37ACE2 (Fig. 2g). We further demonstrated the important role of the RBD in determining receptor use by assessing the entry specificity of pseudotyped viruses containing chimeric viral spike proteins. Bat ACE2 use was changed to hDPP4 use for a chimeric NeoCoV spike containing the MERS-CoV RBD (Fig. 2h). By contrast, a chimeric MERS-CoV spike containing the NeoCoV RBD was inefficient in pseudotyped assembly (Extended Data Fig. 5d–e). Taken together, these results demonstrate that the NeoCoV and PDF-2180 B domains are bona fide RBDs for species-specific interaction with ACE2.

## The structural basis for ACE2 binding

**Fig. 3:**
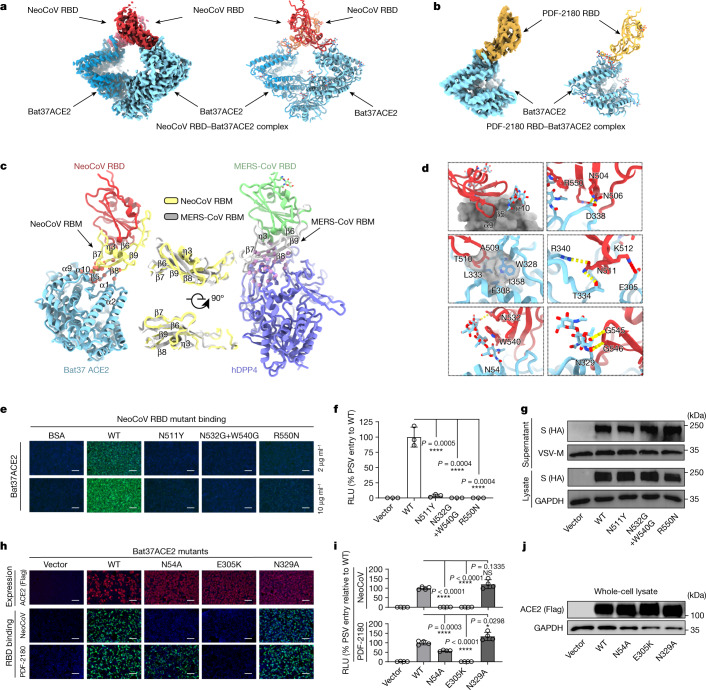
Cryo-EM structures of NeoCoV RBD and PDF-2180 RBD in a complex with Bat37ACE2. **a**,**b**, Cryo-EM density map (left) and ribbon representation (right) of the NeoCoV RBD–Bat37ACE2 complex (**a**) and the PDF-2180 RBD–Bat37ACE2 complex (**b**). NeoCoV RBD, PDF-2180 RBD and Bat37ACE2 are coloured red, orange and cyan, respectively. **c**, Structural comparison between the NeoCoV RBD–Bat37ACE2 complex (left, PDB: 7WPO) and MERS-CoV RBD-hDPP4 complex (right, PDB: 4KR0). The NeoCoV RBD, MERS-CoV RBD, NeoCoV RBM, MERS-CoV RBM, Bat37ACE2 and hDPP4 are coloured red, green, yellow, grey, cyan and purple, respectively. **d**, Magnified view of the NeoCoV RBD–Bat37ACE2 complex interface. All of the structures are shown as ribbon representations with key residues rendered as sticks. Salt bridges and hydrogen bonds are shown as red and yellow dashed lines, respectively. **e**–**g**, The contribution of critical NeoCoV RBD residues to receptor binding (**e**) and pseudotyped virus entry (**f**) in HEK293T-Bat37ACE2 cells. **g**, The effect of mutations on S expression (lysate) and virion incorporation (supernatant) in HEK293T cells. **h**–**j**, The contribution of critical Bat37ACE2 residues on NeoCoV RBD binding (**h**), pseudotyped virus entry (**i**) and the impact of mutations on ACE2 expression (**j**) in HEK293T cells. For **e**–**j**, data are representative of two independent infections assays. Two independent preparations of pseudoviruses were used for the assays shown in **e**–**g**. Data are mean ± s.d. for **f** (biological triplicates of infected cells) and **i** (biological quadruples of infected cells). Statistical analysis was performed using two-tailed unpaired Student’s *t*-tests. For **e** and **h**, scale bars, 100 μm. Source data

**Extended Data Fig. 6:**
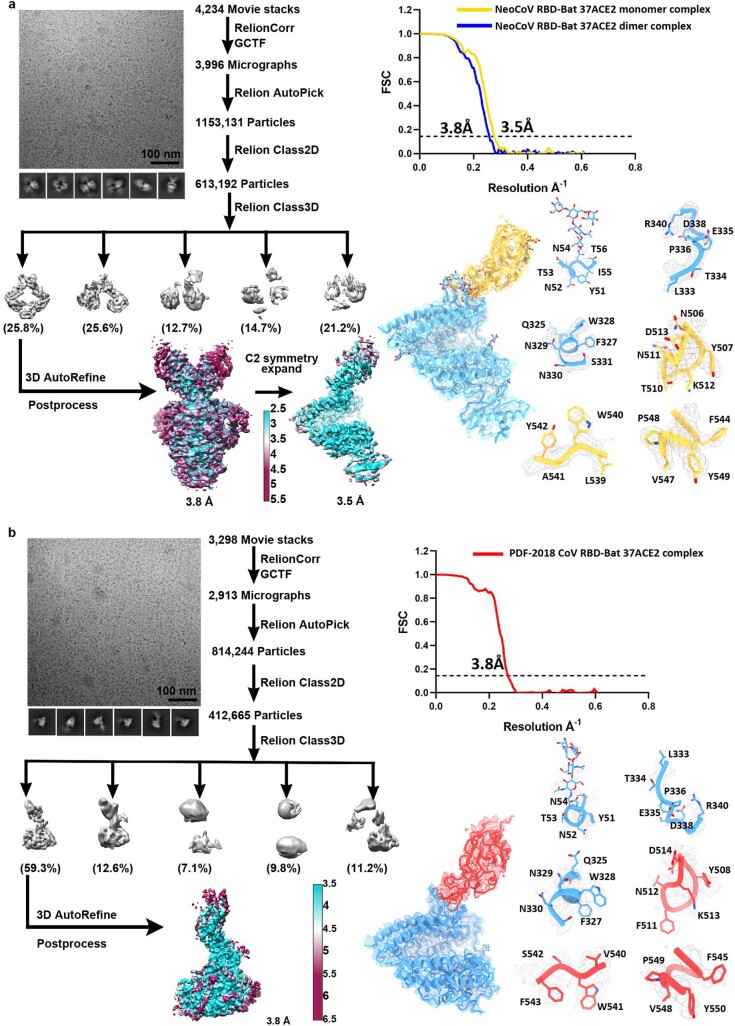
Cryo-EM data processing and validation of the NeoCoV RBD-Bat37ACE2 complex and PDF-2180 RBD-Bat37ACE2 complex cryo-EM datasets. Electron micrograph and flowchart for cryo-EM data processing, resolution estimation of the EM maps, density maps, and atomics models of NeoCoV RBD-Bat37ACE2 complex (**a**) and PDF-2180 RBD-Bat37ACE2 complex (**b**).

**Extended Data Fig. 7:**
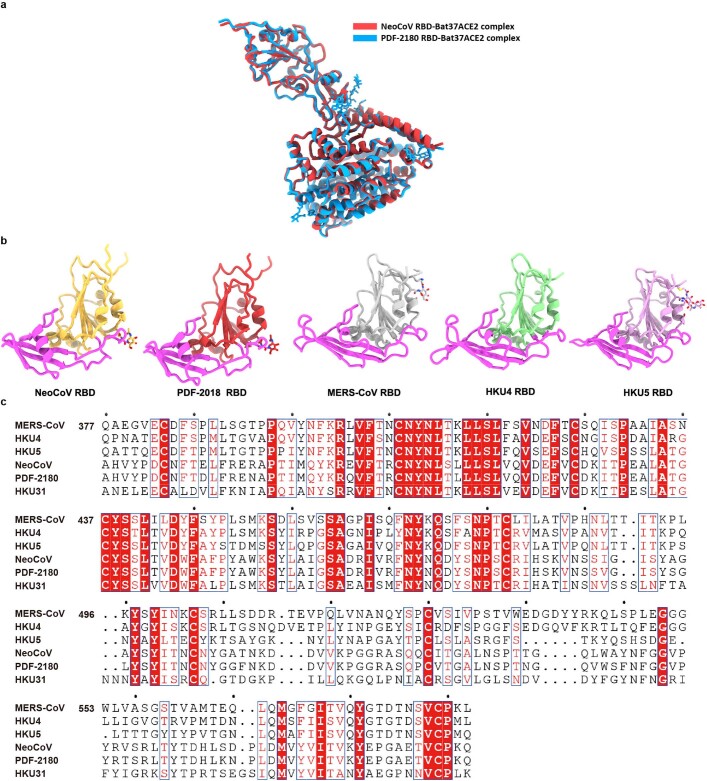
Structural and sequence analysis of different merbecoviruses. **a**, Superimposition of overall structures of NeoCoV RBD-Bat37ACE2 (red) and PDF-2180 RBD-Bat37ACE2 complexes (blue). **b**, Structures of RBDs from different merbecoviruses. MERS-CoV (PDB:4KQZ), HKU4 (PDB:4QZV), HKU5 (PDB:5XGR). **c**, RBD sequence alignments were generated with ClustalW and rendered with ESPript. Identical residues are highlighted with a red background and similar residues are coloured red in blue boxes.

To determine the molecular basis of ACE2 recognition, we performed structural studies of Bat37ACE2 in a complex with the NeoCoV and PDF-2180 RBDs. 3D classification revealed that the NeoCoV RBD–Bat37ACE2 complex primarily adopts a dimeric state with two copies of ACE2 bound to two RBDs, whereas only a monomeric state was observed in the PDF-2180–Bat37ACE2 complex (Figs. 3a,b and Extended Data Fig. 6a,b). We determined the structures of these two complexes at a resolution of 3.8 Å, and *C*_2_ symmetry expansion was performed for the NeoCoV RBD–Bat37ACE2 complex to further improve the resolution to 3.5 Å, enabling a reliable analysis of the interactions involved in binding (Fig. 3a,b, Extended Data Fig. 6a,b and Supplementary Tables 1 and 2). Despite existing in different oligomeric states, the structures revealed that both NeoCoV and PDF-2180 recognize Bat37ACE2 in a very similar manner (Figs. 3a,b and Extended Data Fig. 7a). Thus, we used the NeoCoV RBD–Bat37ACE2 complex structure for detailed analysis. Both RBDs comprise a receptor-binding motif (RBM) and a core subdomain, as described for other betacoronaviruses (Fig. 3c and Extended Data Fig. 7b). The RBM folds as a four-stranded anti-parallel β-sheet (β6–β9) and exposes its tip surface for ACE2 engagement (Fig. 3c). By contrast, the MERS-CoV RBD recognizes the side surface of the DPP4 β-propeller through its four-stranded β-sheet (Fig. 3c). The differences in receptor use for these two viruses can be explained by (1) the local configuration of the NeoCoV RBM, which shows a conformational shift of η3 and β8 disrupting the flat DPP4-binding surface, and (2) the longer MERS-CoV 6–7 and 8–9 loops, relative to NeoCoV, would impair binding to the shallow cavity of bat ACE2 (Fig. 3c and Extended Data Fig. 7b,c).

In the NeoCoV–Bat37ACE2 complex structure, relatively smaller surface areas (498 Å^2^ in NeoCoV RBD and 439 Å^2^ in Bat37ACE2) are buried by the two binding partners compared with their counterparts in the MERS-CoV–DPP4 complex (926 Å^2^ in MERS-CoV RBD and 1,037 Å^2^ in DPP4, using Protein Data Bank (PDB) 4KR0) or the SARS-CoV-2-RBD complex (864 Å^2^ in SARS-CoV-2 RBD and 823 Å^2^ in hACE2, using PDB 6M0J). The NeoCoV RBM inserts into an apical depression constructed by the α9 and α10 helices and a loop connecting α10 and β4 of Bat37ACE2 through a network of electrostatic and hydrophobic interactions involving the tip of the RBM four-stranded β-sheet (Fig. 3d and Supplementary Table 2). Polar interactions are predominantly mediated by residues Asn504, Asn506, Asn511, Lys512 and Arg550 from the NeoCoV RBM and residues Glu305, Thr334, Asp338 and Arg340 from Bat37ACE2 (Fig. 3d and Supplementary Table 2). Moreover, the methyl groups of the NeoCoV RBM Ala509 and Thr510 residue side chains are packed into a hydrophobic pocket formed by residues Phe308, Trp328 and Leu333 of Bat37ACE2. The PDF-2180 RBM contains a T510F substitution relative to NeoCoV, which further improves hydrophobic interactions with Bat37ACE2 and is consistent with the increased binding affinity observed for this NeoCoV point mutant (Figs. 3d and Supplementary Table 2). Moreover, the Bat37 ACE2 glycans at positions Asn54 and Asn329 sandwich the strands (β8–β9), forming extensive interactions with RBD residue Trp540 and to a lesser extent with Asn532, Leu539, Ala541, Gly545, Gly546 and Val547 from the NeoCoV RBD, underpinning virus–receptor associations (Fig. 3d and Supplementary Table 2).

The importance of key interface residues was verified by mutagenesis and assessment of receptor binding and pseudotyped virus entry. As expected, the mutations N511Y and R550N in the NeoCoV RBD, which abolish the polar contacts and introduce steric clashes, resulted in a significant decrease in RBD binding and viral entry (Fig. 3e–g). Similarly, the E305K mutation in Bat37ACE2, which eliminates a salt bridge, also impaired receptor function. Moreover, the Bat37ACE2 N54A mutation (abolishing the *N*-glycosylation) and NeoCoV W540G/N532G substitutions (eliminating interactions with the glycan) hindered binding. These results confirmed the importance of the protein–glycan interactions identified in viral–receptor recognition, as recently described for the human-infecting CCoV-HuPn-2018^30^. By contrast, the mutation N329A, which abolishes the *N*-glycosylation at site Asn329, did not have a major effect on receptor function (Fig. 3h–j).

## Determinants of human ACE2 recognition

**Fig. 4:**
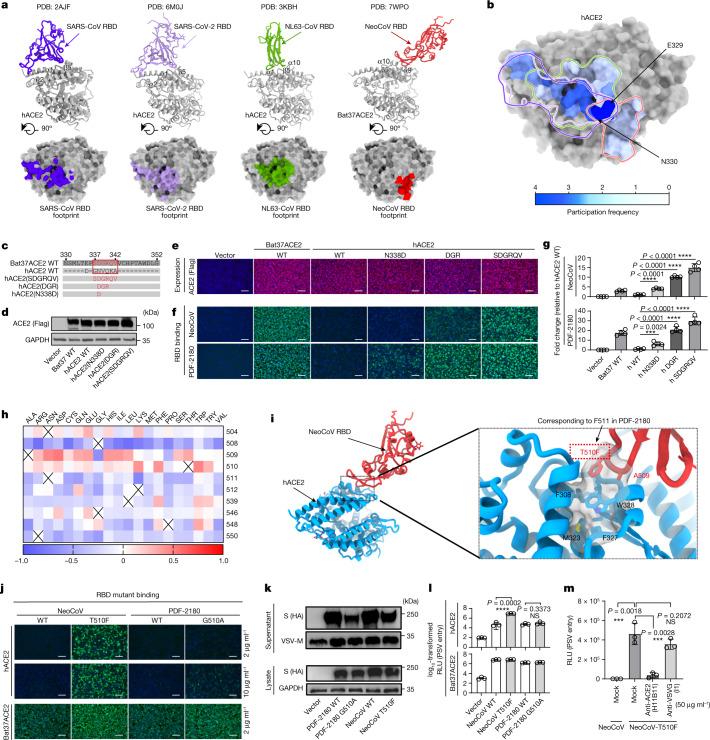
Molecular determinants affecting hACE2 recognition by NeoCoV and PDF-2180. **a**, RBD binding modes and receptor (hACE2 or Bat37ACE2) footprints of the four indicated ACE2-using coronaviruses. **b**, The overlap of the ACE2 footprints. The heat map indicates the per-residue frequency of participation to the virus–ACE2 interfaces identifying residues 329–330 as a virus-binding hot spot. **c**, Schematic of the hACE2 swap mutants with Bat37ACE2 counterparts. **d**,**e**, Expression levels of hACE2 mutants were analysed using western blotting (**d**) and immunofluorescence (**e**). h, human. **f**,**g**, The receptor function of hACE2 mutants was evaluated by NeoCoV RBD binding (**f**) and NeoCoV S pseudotyped virus entry in HEK293T-hACE2 (h-WT) cells (**g**). The fold changes of pseudotyped virus entry relative to wild-type hACE2 are shown. For **e** and **f**, scale bars, 100 μm. **h**, Mutations in the interaction between NeoCoV RBD and Bat37ACE2 were taken as the input of mCSM-PPI2 to predict the change of free binding energy (ΔΔ*G*, kcal mol^−1^). Mutations with lower ΔΔ*G* are marked in blue and those with higher ΔΔ*G* are marked in red. **i**, Computational modelling of the NeoCoV RBD–hACE2 complex obtained by superposing the hACE2 structure (PDB: 6M0J, blue) on the Bat37ACE2-bound NeoCoV RBD (red) structure described here. Magnified view of the T510F NeoCoV RBD mutation. **j**–**l**, The effect of NeoCoV and PDF-2180 RBM mutations on hACE2 recognition assessed by RBD–hFc binding (**j**), spike expression (lysate) and virion incorporation (supernatant) (**k**), and pseudotyped virus entry efficiency (**l**) in HEK293T-hACE2 and HEK293T-Bat37ACE2 cells. For **j**, scale bars, 100 μm. **m**, hACE2-dependent entry of NeoCoV-T510F S pseudotyped virus in Caco-2 cells in the presence of 50 μg ml^−1^ anti-ACE2 (H11B11) or anti-VSVG (I1) antibodies. Mock, no antibody. For **d**–**g** and **j**–**l**, data are representative of two infection assays with independent transfections. For **m**, data are representative of two infection assays. Data are mean ± s.d. for **g** (*n* = 4 biologically independent cells) and **l** and **m** (*n* = 3 biologically independent cells). Statistical analysis was performed using two-tailed unpaired Student’s *t*-tests. Source data

As mentioned above, the NeoCoV and PDF-2180 RBDs cannot efficiently interact with hACE2. Here we first examined the molecular determinants that restrict hACE2 from supporting the entry of these two pseudotyped viruses. By comparing the binding interface of the other three hACE2-using coronaviruses, we found that the SARS-CoV, SARS-CoV-2 and NL63 RBDs share overlapping footprints on hACE2 that barely overlapped with the region engaged by NeoCoV (Fig. 4a). Indeed, only residues 329–330 are in contact with all four virus RBDs (Fig. 4b). On the basis of sequence alignment and structural analysis of hACE2 and Bat37ACE2, we predicted that inefficient hACE2 binding could be attributed to suboptimal residues at the binding interfaces with NeoCoV and PDF-2180, especially around the divergent residues around 337–342 (Fig. 4c). To test this hypothesis, we replaced these hACE2 residues with those from the Bat37ACE2 orthologue (Fig. 4c,d). The mutations led to a significant gain of binding affinity with RBDs relative to wild-type hACE2, and an approximately 15-fold and 30-fold increase in the entry efficiency of NeoCoV and PDF-2180 S pseudotyped viruses, respectively (Fig. 4e–g). These results demonstrate that this region is critical for receptor engagement and host range determination, with residue Asn338 having a crucial role in restricting human receptor use, consistent with the intensive interactions that it forms with the NeoCoV RBD (Fig. 3d).

**Extended Data Fig. 8:**
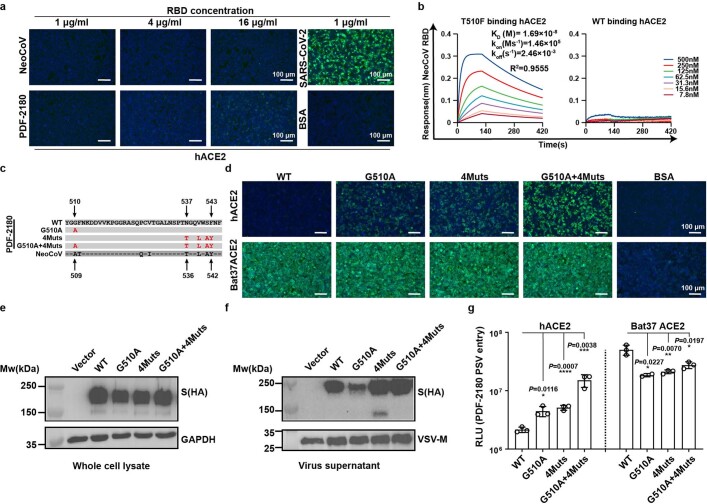
Characterization of NeoCoV and PDF-2180 RBM mutations enhancing human ACE2 binding and pseudovirus entry. **a**, Analysis of binding of various concentrations of the NeoCoV and PDF-2180 RBD-hFc to HEK293T cells stably expressing hACE2. The SARS-CoV-2 RBD-hFc was used as a positive control. Mock indicates no protein added during protein incubation. Data representative of two live cell binding assays using independent preparations of RBD-hFc proteins. **b**, Analysis of binding kinetics of the interaction between NeoCoV RBD WT or T510F with hACE2 using BLI. Reported K_D_ values correspond to avidities due to the utilization of dimeric ACE2 constructs. Representative of two independent experiments. Unfitted curves can be found in Supplementary Figure 4b. **c**, Identification of PDF-2180 S mutations enhancing hACE2 binding. Sequence alignment of the NeoCoV and PDF-2180 RBMs and definition of the PDF-2180 mutants generated. **d**, Binding of PDF-2180 RBD-hFc mutants to hACE2- or Bat37ACE2-expressing HEK293T cells. Data presented were performed in two independent assays with similar results. **e**, Western blot analysis of the expression levels of PDF-2180 S protein mutants in HEK293T cells. **f**, Western blot analysis of the packaging efficiency of PDF-2180 S mutants in VSV pseudotyped viruses. **g**, Entry efficiency of PDF-2180 S mutant pseudotyped viruses into hACE2- or Bat37ACE2- expressing HEK293T cells. Experiments presented were independently performed twice with similar results for **d**-**g**. Representative data of **g** are presented as mean ± SD (n = 3 biological triplicates). Two-tailed unpaired Student’s *t*-test; *P < 0.05,**P < 0.01; ***P < 0.005, and ****P < 0.001 Source data

To assess the risk of NeoCoV and PDF-2180 adaptation to hACE2, we sought to identify the RBM amino acid changes that would enable more efficient engagement of hACE2 based on our cryo-EM structures assisted by the mCSM-PPI2 software (Fig. 4h). We predicted that increasing hydrophobicity around the residue Thr510 of NeoCoV might enhance interactions with hACE2 (Fig. 4i). The PDF-2180 spike equivalent residue is Phe511, which is consistent with its slightly higher affinity for hACE2 (Extended Data Fig. 8a). Indeed, the NeoCoV T510F RBD mutant had a substantially increased binding affinity for hACE2 (*K*_D,app_ = 16.9 nM), and the corresponding pseudotyped virus entered HEK293T-hACE2 cells markedly better than the wild-type pseudotyped virus (Fig. 4j–l and Extended Data Fig. 8b). However, the PDF-2180 S pseudotyped virus entered these cells with much lower efficiency than the NeoCoV T510F S pseudotyped virus, indicating that other RBM residues might restrict efficient interactions with hACE2. Indeed, a G to A mutation at site 510 (corresponding to A509 in NeoCoV), increasing the local hydrophobicity, marginally enhanced PDF-2180 RBD binding to hACE2 (Fig. 4j–l). Additional replacement of residues 537–543 of the PDF-2180 spike with those of the NeoCoV spike further increased PDF-2180 binding affinity for hACE2 (Extended Data Fig. 8c–g). Moreover, the NeoCoV T510F S pseudotyped virus entered human colon cell line Caco-2 with much higher efficiency than wild-type NeoCoV, and was inhibited by the ACE2-targeting antibody H11B11^31^ (Fig. 4m).

## Spike architecture and antigenicity

**Fig. 5:**
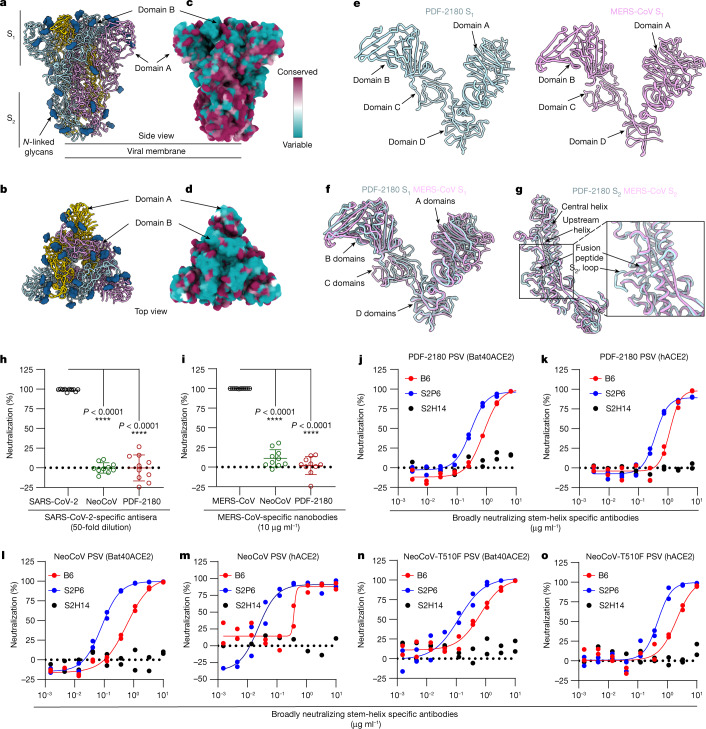
The architecture and antigenicity of the PDF-2180 spike glycoprotein. **a**,**b**, Ribbon diagram of the cryo-EM structure of PDF-2180 S ectodomain trimer viewed along two orthogonal orientations (side (**a**) and top (**b**)). **c**,**d**, Sequence conservation between PDF-2180 S and the spikes of different isolates of MERS-CoV plotted on the PDF-2180 S structure viewed from the side (**c**) and top (**d**). Conservation analysis was performed using Consurf^51^. **e**, Ribbon diagram of the PDF-2180 (left) and MERS-CoV (right) S_1_ subunits. **f**, Superimposition of the PDF-2180 and MERS-CoV S_1_ subunits. **g**, Ribbon diagram of the superposed PDF-2180 and MERS-CoV S_2_ subunits highlighting the similarity of the fusion machinery. Inset: magnified view of the fusion peptide region. **h**, Neutralizing activity of CoronaVac vaccine-elicited (three doses) human sera against SARS-CoV-2, NeoCoV and PDF-2180 S pseudotyped viruses. **i**, Neutralizing activity of MERS-CoV RBD-targeting nanobodies against MERS-CoV, NeoCoV and PDF-2180 S pseudotyped viruses^38,39^. HEK293T-hACE2 cells for SARS-CoV-2; HEK293T-hDPP4 cells for MERS-CoV; HEK293T-Bat37ACE2 cells for NeoCoV and PDF-2180. For **h** and **i**, data are mean ± s.d. *n* = 10 sera or antibodies. Each point represents the mean neutralization value of three biologically independent infection replicates. Statistical analysis was performed using two-tailed paired Student’s *t*-tests. The inhibitory efficiency of specific samples is summarized in Extended Data Fig. 10b,c. **j**–**o**, PDF-2180 (**j**,**k**), NeoCoV (**l**,**m**) and NeoCoV-T510F S (**n**,**o**) pseudotyped viruses entry in the presence of the indicated dilutions of B6 (red) and S2P6 (blue) antibodies, and a SARS-CoV-2-RBD-specific IgG (S2H14, black) was used as negative control. HEK293T cells transiently transfected with Bat40ACE2 (**j**,**l**,**n**) or hACE2 (**k**,**m**,**o**) were used as target cells. For **j**–**o**, the average of technical duplicates (one representative experiment out of two independent experiments, that is, a biological duplicate) is shown. For **h**–**o**, data are representative of two independent neutralization assays. Source data

**Extended Data Fig. 9:**
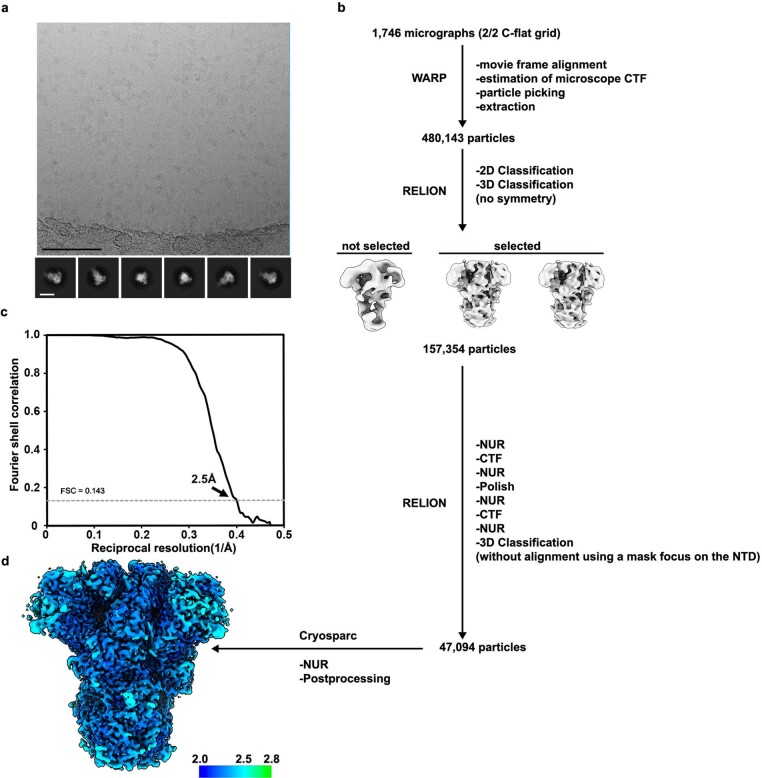
Cryo-EM data processing and validation of the PDF-2180 S cryo-EM dataset. **a**, Representative electron micrograph (top) and class averages (bottom) of PDF-2180 S embedded in vitreous ice. Scale bar of the micrograph: 1000 Å. Scale bar of the class averages: 100 Å. **b**, Flowchart for cryo-EM data processing of PDF-2180 S trimer. **c**, Gold-standard Fourier shell correlation curve for the PDF-2180 S trimer. The 0.143 cut-off is indicated by a horizontal dashed grey line. **d**, Local resolution map for the PDF-2180 spike trimer.

**Extended Data Fig. 10:**
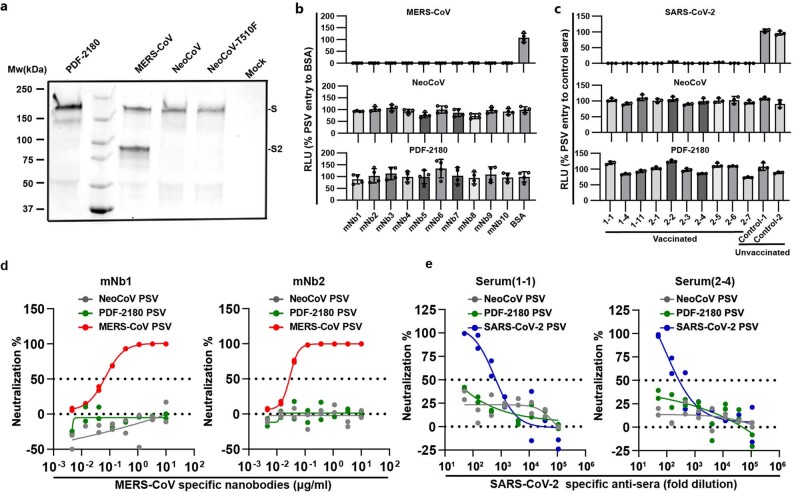
S_1_/S_2_ cleavage and antibody neutralization efficiency of PDF-2180, NeoCoV, NeoCoV-T510F mutant, or MERS-CoV S pseudotyped viruses. **a**, Western blot of VSV pseudotyped particles harbouring PDF-2180, NeoCoV, NeoCoV-T510F mutant or MERS-CoV S glycoproteins (detected using the B6 antibody) used for the B6 and S2P6 neutralization assays. Experiments were performed in duplicates, and representative data were shown. Mw: molecular weight ladder. Full-length S and S_2_ subunit bands are indicated on the right-hand side of the blot. **b**-**c**, Inhibitory activity of 10 MERS-CoV RBD specific nanobodies at 10 μg/ml (**b**) and 10 SARS-CoV-2 specific anti-sera or two unvaccinated control sera at 50-fold dilution (**c**). Data are presented as mean ± SD (biological triplicates of two independent experiments). **d**-**e**, Dose-dependent inhibition of the entry of the indicated pseudotyped viruses by representative MERS-CoV RBD specific nanobodies (**d**) and SARS-CoV-2 specific anti-sera (**e**). Data are presented as mean (n = 2 biological duplicates), representative of two independent experiments. Source data

To understand the antigenic landscape of these ACE2-using bat merbecoviruses and provide a blueprint for vaccine design, we determined a cryo-EM structure of the prefusion PDF-2180 spike trimer. 3D classification of the data revealed the presence of a single conformation corresponding to a closed trimer for which we determined a structure at a resolution of 2.5 Å (Fig. 5a,b, Extended Data Fig. 9a–d and Supplementary table 1). The PDF-2180 S_1_ and S_2_ subunits are similar to the MERS-CoV equivalent subunits with which they can be superimposed with a root‐mean‐square distance of 2.8 Å and 1.4 Å for 566 and 443 aligned residues, respectively, reflecting their degree of sequence conservation (Fig. 5c–g). Although both spikes contain a furin-cleavage site at the S_1_–S_2_ junction, processing during synthesis was detected for MERS-CoV but not for PDF-2180 S pseudotyped virions (Extended Data Fig. 10a). Moreover, the S_1_–S_2_ cleavage site is resolved in the PDF-2180 S ectodomain trimer, but not MERS-CoV S, reflecting the different accessibility of this motif and putatively contributing to the closed conformation of the RBDs^32^ (Fig. 5g). Differential S cleavage has previously been shown to modulate plasma membrane versus endosomal fusion and suggests that these two viruses might use different host cell entry pathways besides their distinct receptor usage^33–35^.

Previous research indicated that the RBD-directed LCA60 MERS-CoV neutralizing antibody does not neutralize a MERS-CoV virus chimera containing the PDF-2180 spike, in agreement with the aforementioned structural differences between the RBMs of these two viruses^28,36,37^. Furthermore, no inhibition of the PDF-2180 or NeoCoV S pseudotyped viruses was observed with SARS-CoV-2 antisera obtained from individuals who were vaccinated with inactivated SARS-CoV-2 vaccine or a panel of ten RBD-neutralizing MERS-CoV nanobodies^38,39^ (Fig. 5h,i and Extended Data Fig. 10b–e). As the SARS-CoV-2 S_1_ subunit, especially the RBD, accounts for most of the infection- or vaccine-elicited serum neutralizing activity^40–42^ and several potent MERS-CoV neutralizing antibodies target the RBD, our findings suggest that immunity to either SARS-CoV-2 or MERS-CoV would not elicit appreciable titres of cross-neutralizing antibodies owing to marked divergence of their RBDs^28,36,43^.

We previously described the B6 and S2P6 cross-reactive and broadly neutralizing betacoronavirus antibodies targeting a conserved stem helix epitope in the spike S_2_ fusion machinery^44,45^. As the stem helix is strictly conserved between MERS-CoV, PDF-2180 and NeoCoV S, we assessed the ability of these two monoclonal antibodies to block PDF-2180 and NeoCoV S-mediated entry. B6 and S2P6 inhibited entry of PDF-2180, NeoCoV and NeoCoV-T510F S pseudotyped viruses into HEK293T cells that were transiently transfected with Bat40ACE2 (*A. pallidus)* or hACE2 (Fig. 5j–o) in a dose-dependent manner. Moreover, the strict architectural conservation of the fusion peptide region between PDF-2180 and MERS-CoV (Fig. 5g) explains that recently described monoclonal antibodies targeting this motif neutralize PDF-2180 S pseudovirus as well^46^. Collectively, these data underscore that the conservation of the fusion machinery between NeoCoV, PDF-2180 and MERS-CoV S is associated with the retention of inhibitory activity of several stem-helix- and fusion-peptide-directed broadly neutralizing betacoronavirus antibodies, some of which have been shown to reduce the viral burden in small-animal challenge models^45,47^.

## Discussion

The lack of knowledge of the receptors used by many bat coronaviruses limits our understanding of their entry and interspecies transmission mechanisms. We show that close relatives of MERS-CoV, such as NeoCoV and PDF-2180, engage bat ACE2 for efficient cellular entry. However, HKU5 and EriCoV do not use bat DPP4 or hedgehog ACE2 for entry, highlighting the complexity of receptor use by coronaviruses.

Many sarbecoviruses, alphacoronavirus NL63 and a group of merbecoviruses reported in this study share ACE2 for cellular entry. Our structural analysis indicates that NeoCoV and PDF-2180 bind to an apical side surface of ACE2, which is largely different from the surface engaged by other ACE2-using coronaviruses (Fig. 4a). The different interaction modes of the three groups of ACE2-using coronaviruses along with the distinct structures of their RBMs or receptor-binding loops suggest a convergent acquisition of ACE2 use during evolution^21^. The evolutionary advantage of ACE2 use for different coronaviruses remains unclear and deserves further investigation.

Our results support the previous hypotheses that the origin of MERS-CoV might be a result of an intraspike recombination event between a NeoCoV-like virus and a DPP4-using virus^26^. RNA recombination can occur during the co-infection of different coronaviruses, giving rise to a new virus with different receptor use and host tropism^48^. It remains unclear whether the event took place in bats, camels or other hosts, and when the host switching would have happened. Although bat merbecoviruses are geographically widespread, the two known ACE2-using merbecoviruses were both discovered in Africa. Considering that both NeoCoV and PDF-2180 S glycoproteins mediate relatively inefficient entry into human cells, the acquisition of the hDPP4-binding domain or the S_1_ subunit in an unknown host coinfected with a DPP4-using virus might have driven the emergence of MERS-CoV. Further studies will be necessary to determine the evolutionary trajectory of MERS-CoV.

The host range determinants on ACE2 are primary barriers for cross-species transmission of these viruses. Our results show that NeoCoV and PDF-2180 favour ACE2 from Yangochiropteran bats, especially vesper bats (Vespertilionidae), which their host belongs to, but not ACE2 orthologues from Yinpterochiropteran bats. To date, most merbecoviruses were found in species belonging to the Vespertilionidae group, a highly diverse and widely distributed family^8^. Although the two viruses could not use hACE2 efficiently, our study also reveals that single-residue substitution increasing local hydrophobicity around NeoCoV site 510 could enhance their affinity for hACE2 and enable them to enter human cells endogenously expressing ACE2. Considering the extensive mutations in the RBD regions of the SARS-CoV-2 variants, especially the heavily mutated omicron variant, these viruses may hold a latent potential to infect humans by further adaptation through mutations^49,50^. However, zoonotic spillover is a complex process involving multiple factors other than receptor recognition, including proteolytic spike activation, replication, immune response and contact opportunity. To date, there is no evidence that NeoCoV or PDF-2180 can infect mammals other than bats. Our results also did not confirm that NeoCoV carrying T510F, which has not been found in nature, can infect humans in vivo.

Although investigations using authentic viruses will support further evaluation of their risk for humans, the biosafety concern of artificially altering viral tropism should be taken seriously as antibodies elicited by current COVID-19 vaccination and anti-MERS-CoV RBD antibodies are inadequate to neutralize PDF-2180 and NeoCoV. Our results showed that broadly neutralizing betacoronavirus antibodies targeting the conserved stem helix retained their activity in inhibiting the entry of NeoCoV and PDF-2180. Thus, the clinical advancement of these antibodies could therefore enable therapeutic deployment in case of zoonotic spillover of these viruses.

Overall, we identified the functional receptor of a potential MERS-CoV ancestor in bats, which unexpectedly turned out to be ACE2, thereby enabling in-depth research of these important viruses presenting a possible risk of zoonotic emergence. Our study adds to the knowledge about the complex receptor use of coronaviruses, highlighting the importance of surveillance and research on these viruses to prepare for potential future outbreaks.

## Methods

### Receptor and virus sequences

The acquisition of sequences of 46 bat ACE2 and hACE2 was described in our previous study^29^. The five bat DPP4 and hDPP4 sequences were directly retrieved from GenBank (human DPP4, NM_001935.3; Bat37, *P. pipistrellus*, KC249974.1) or extracted from whole genome sequence assemblies of the bat species retrieved from GenBank (Bat25, *Sturnira hondurensis*, GCA_014824575.2; Bat29, *Mormoops blainvillei*, GCA_004026545.1; Bat36, *Aeorestes cinereus*, GCA_011751065.1; Bat40, *A. pallidus*, GCA_007922775.1). The whole genome sequences of different coronaviruses were retrieved from GenBank. Accession numbers are as follows: MERS-CoV (NC_019843.3), Camel MERS-CoV KFU-HKU 19Dam (KJ650296.1), HKU4 (NC_009019.1), HKU5 (NC_009020.1), ErinaceusCoV/HKU31 strain F6 (MK907286.1), NeoCoV (KC869678.4), PDF-2180 (NC_034440.1), ErinaceusCoV/2012-174 (NC_039207.1), BtVs-BetaCoV/SC2013 (KJ473821.1), BatCoV/*H.* *savii*/Italy (MG596802.1), BatCoV HKU25 (KX442564.1), BatCoV ZC45 (MG772933.1), SARS-CoV-2 (NC_045512.2), NL63 (JX504050.1) and 229E (MT797634.1). All receptor and viral gene sequences used in this study were commercially synthesized by Genewiz, GenScript or GeneArt. The sources, accession numbers and sequences of the receptors and viruses are summarized in Supplementary Table 4.

### Collection of SARS-CoV-2 antisera

All of the vaccinated sera were collected from volunteers at about 21 days after the third dose of the WHO-approved inactivated SARS-CoV-2 vaccine (CoronaVac, Sinovac). The median age of volunteers was 37 years. A total of 44% of participants were male and 56% were female. All of the volunteers were recruited by Sinovac. None of the participants had a history of previous SARS-CoV-2 infection, and none reported serious adverse events after vaccination. All of the volunteers were provided informed written consent forms, and the whole study was conducted according to the requirements of Good Clinical Practice of China. The procedures about human participants were approved by the Ethics Committee (seal) of Beijing Youan Hospital, Capital Medical University with an approval number of LL-2021-042-K.

### Bioinformatic and computational analyses

Protein sequence alignment was performed using the MUSCLE algorithm by MEGA-X software (v.10.1.8) or ClustalW (https://www.genome.jp/tools-bin/clustalw). For phylogenetic analysis, nucleotide or protein sequences of the viruses were first aligned using the ClustalW and the MUSCLE algorithm, respectively. Phylogenetic trees were subsequently generated using the maximal-likelihood method in MEGA-X (1,000 bootstraps). The model and the other parameters used for phylogenetic analysis were applied following the recommendations after finding the best DNA/protein models using the software. The nucleotide similarity of coronaviruses was analysed using SimPlot (v.3.5.1) with a sliding windows size of 1,000 nucleotides and a step size of 100 nucleotides using gap-stripped alignments and the Kimura (two-parameter) distance model. Molecular dynamics prediction of the effect of residue mutations on protein–protein interactions was conducted by mCSM-PPI2 (http://biosig.unimelb.edu.au/mcsm_ppi2/)^52^.

### Plasmids

Human codon-optimized sequences of various receptors and their mutants were cloned into a lentiviral transfer vector (pLVX-EF1a-Puro, Genewiz) with a C-terminal 3×Flag tag (DYKDHD-G-DYKDHD-I-DYKDDDDK). The DNA sequences of human codon-optimized NeoCoV S (GenBank: AGY29650.2), PDF-2180 S (GenBank: YP_009361857.1), HKU4 S (GenBank: AWH65899.1), HKU5 S (GenBank: YP_001039962.1), HKU31 S (GenBank: QGA70692.1), SARS-CoV-2 S (GenBank: YP_009724390.1) and MERS-CoV S (GenBank: YP_009047204.1) were cloned into the pCAGGS vector or pcDNA3.1(−) vector with a deletion of the last 13–15 residues (or 18 amino-acids in the SARS-CoV-2 S construct) or replacement by an HA tag (YPYDVPDYA) for higher VSV pseudotyping efficiency^53^. Plasmids expressing coronavirus RBD–IgG-hFc fusion proteins were generated by inserting the coding sequences of NeoCoV RBD (380–585), PDF-2180 RBD (381–586), HKU4 (382–593), HKU5 RBD (385–586), HKU31 RBD (366–575), SARS-CoV-2 RBD (331–524) and MERS-CoV RBD (377–588) into the pCAGGS vector with an N-terminal CD5 secretion leading sequence (MPMGSLQPLATLYLLGMLVASVL) and C-terminal hFc tag for easy purification and detection. Plasmids expressing soluble bat and human ACE2 proteins (corresponding to residues 18–740 in hACE2) were constructed by inserting the ectodomain-coding sequences (containing a collectrin-like dimerization domain) into the pCAGGS vector with an N-terminal CD5 leading sequence and a C-terminal twin-strep tag and 3×Flag tandem sequences (WSHPQFEKGGGSGGGSGGSAWSHPQFEK-GGGRS-DYKDHDGDYKDHDIDYKDDDDK). Virus S proteins or receptor mutants or chimeras were generated by overlapping PCR. For DSP-based cell–cell fusion assays, the dual reporter split proteins were expressed by pLVX-EF1a-Puro-based plasmids carrying the rLucN(1–155)-sfGFP1–7(1–157) and sfGFP8–11(158–231)-rLucC(156–311) coding sequences (summarized in Supplementary Table 4), which were constructed in the laboratory based on a previous study^54,55^. Plasmids expressing codon-optimized anti-ACE2 antibodies (H11B11; GenBank MZ514137 and MZ514138)^31^, B6^44^, S2P6^45^ and S2H14^40^ were constructed by inserting the heavy-chain and light-chain coding sequences into the pCAGGS vector with N-terminal CD5 leader sequences, respectively. For anti-MERS-CoV nanobody–hFc fusion proteins, nanobody coding sequences were synthesized and cloned into the pCAGGS vector with N-terminal CD5 leader sequences and C-terminal hFc tags^38,39^ (Supplementary Table 4). MERS-CoV-specific nanobodies and H11B11 coding sequences were synthesized by Sangon Biotech or Tsingke Biotechnology. For S trimer cryo-EM analysis, PDF-2180 S full-length gene (GenBank: YP_009361857.1) was synthesized by GeneArt, codon-optimized for expression in mammalian cells, cloned into pcDNA3.1(−) between XbaI and BamHI in frame with a Kozak’s sequence to direct translation and with the endogenous signal peptide. PDF-2180 S ectodomain gene was derived from the S full-length construct and comprised residues 1 to 1,286 followed by a foldon trimerization domain and a C-terminal His tag to assist purification. The full-length gene for expression of *A. pallidus* ACE2 (QJF77789) used in pseudotyped virus assays was synthesized by GeneScript and cloned in pCDNA3(+). The full-length gene for expressing hACE2 was described in one of our previous studies^41^.

### Cell lines

HEK293T (CRL-3216), Vero E6 cells (CRL-1586), A549 (CCL-185), BHK-21 (CCL-10), Caco-2 (HTB-37) and the bat epithelial cell line Tb 1 Lu (CCL-88) were purchased from American Type Culture Collection (ATCC). The human hepatocellular carcinoma cell line Huh-7 (SCSP-526) were obtained from the Cell Bank of Type Culture Collection, Chinese Academy of Sciences. All these cells were cultured in Dulbecco’s modified Eagle medium, (DMEM, Monad) supplemented with 10% fetal bovine serum (FBS), 2.0 mM of l-glutamine, 110 mg l^−1^ of sodium pyruvate and 4.5 g l^−1^ of d-glucose. An I1-hybridoma (CRL-2700) cell line secreting a neutralizing mouse monoclonal antibody against the VSV glycoprotein (VSVG) was cultured in minimum essential medium with Earles’s balanced salts solution, 2.0 mM l-glutamine (Gibco) and 10% FBS. All cells were cultured at 37 °C in 5% CO_2_ with regular passaging every 2–3 days. HEK Expi 293F cells (A14527, Thermo Fisher Scientific) expressing protein for cryo-EM analysis was cultured in OPM-293 CD03 serum-free medium (OPM, Shanghai OPM Biosciences).

### Stable cell lines

Stable cell lines overexpressing different receptors were generated by lentivirus transduction and antibiotic selection. Specifically, the lentiviruses carrying the target genes were produced by co-transfection of lentiviral transfer (pLVX-EF1a-Puro,) and packaging plasmids pMD2G (Addgene, 12259) and psPAX2 (Addgene, 12260) into HEK293T cells using Lip2000 transfection reagent (Biosharp, BL623B). The lentivirus-containing supernatant was collected and pooled at 24 and 48 h after transfection. Cells were transduced by the lentivirus after 16 h in the presence of 8 μg ml^−1^ polybrene. Stable cells were selected and maintained in the growth medium with puromycin (1 μg ml^−1^). Cells selected for at least 10 days were considered to be stable cell lines and were used in various experiments.

### Protein expression and purification

Antibodies, nanobody–hFc, soluble ACE2 ectodomain and RBD–hFc fusion proteins were expressed in HEK293T cells by transfecting the corresponding plasmids with GeneTwin reagent (Biomed, TG101-01) according to the manufacturer’s instructions. Then, 4 h after transfection, the culture medium of the transfected cells was replenished by SMM 293-TII Expression Medium (Sino Biological, M293TII). Protein-containing supernatants were collected every 2–3 days. Antibodies, nanobody–hFc and recombinant RBD–hFc proteins were captured by Pierce Protein A/G Plus Agarose (Thermo Scientific, 20424), washed with wash buffer (100 mM Tris/HCl, pH 8.0, 150 mM NaCl, 1 mM EDTA), eluted with pH 3.0 glycine buffer (100 mM in H_2_O) and then immediately balanced by 1/10 volume of UltraPure 1 M Tris-HCI, pH 8.0 (15568025, Thermo Fisher Scientific). The twin-strep-tag-labelled proteins were captured by Strep-Tactin XT 4Flow high-capacity resin (IBA, 2-5030-002), washed with washing buffer and eluted with buffer BXT (100 mM Tris/HCl, pH 8.0, 150 mM NaCl, 1 mM EDTA, 50 mM biotin). Eluted proteins were concentrated and buffer-changed to PBS through ultrafiltration. Protein concentrations were determined using the Omni-Easy Instant BCA Protein Assay Kit (Epizyme, ZJ102). Purified proteins were aliquoted and stored at −80 °C. For cryo-EM analysis, NeoCoV RBD(380–588), PDF-2018 RBD(381–589) and Bat37ACE2(18–740) were synthesized and subcloned into the pCAGGS vector with a C-terminal twin-strep tag. In brief, these proteins were expressed by transient transfection of HEK Expi 293F cells (Gibco, Thermo Fisher Scientific, A14527) using polyethylenimine MAX (MW 40,000; Polysciences) or 293-free Transfection Reagent (Thermo Fisher Scientific). After 4 days, the supernatant was collected and cells were kept in culture for an additional 4 days, yielding two collections per transfection. The RBD and ACE2 protein samples were further purified by size-exclusion chromatography using a Superdex 75 10/300 Increase column (GE Healthcare) or a Superdex 200 10/300 Increase column (GE Healthcare) in 20 mM HEPES, 100 mM NaCl, pH  7.5. For the RBD–receptor complex (NeoCoV RBD–Bat37ACE2/PDF-2180 RBD–Bat37ACE2), NeoCoV RBD or PDF-2180 RBD was mixed with Bat37ACE2 at the ratio of 1.2:1, and incubated for 30 min on ice. The mixture was then processed for gel-filtration chromatography. The fractions containing the complex were collected and concentrated to 2 mg ml^−1^.

For PDF-2180 S ectodomain expression, HEK293F cells were grown in suspension, with rotation at 130 rpm, in FreeStyle 293 Expression Medium (Life Technologies) at 37 °C in a humidified 8% CO_2_ incubator. The wild-type PDF-2180 S ectodomain construct was transfected into 500 ml cultures with cells grown to a density of 1 million cells per ml using 293-free Transfection Reagent (Thermo Fisher Scientific). After 4 days, the supernatant was collected and cells were kept in culture for an additional 4 days, yielding two collections per transfection. The supernatants were clarified by centrifugation at 800*g* for 10 min, supplemented with 350 mM NaCl and 25 mM Tris-HCl pH 8.0, further centrifuged at 14,000*g* for 30 min and passed through a 1 ml His trap HP column (Cytiva) that had been equilibrated with binding buffer (25 mM Tris pH 7.4 and 350 mM NaCl). The PDF-2180 S ectodomain was eluted using a linear gradient of 500 mM imidazole. Purified protein was concentrated, buffer-exchanged into Tris-saline buffer (25 mM Tris pH 8, 150 mM NaCl) and quantified using absorption at 280 nm. Purified S glycoprotein was concentrated, and flash-frozen before negative staining and cryo-EM analysis.

### Cryo-EM sample preparation and data collection

For cryo-EM sample preparation, the NeoCoV RBD–Bat37ACE2 or PDF-2018 RBD–Bat37ACE2 complexes were diluted to 0.5 mg ml^−1^. Holy-carbon gold grids (Cflat R1.2/1.3 mesh 300) were freshly glow-discharged with a Solarus 950 plasma cleaner (Gatan) for 30 s. A 3 μl aliquot of the mixture complex was transferred onto the grids, blotted with filter paper at 16 °C and 100% humidity, and plunged into the ethane using the Vitrobot Mark IV (FEI). For these complexes, micrographs were collected at 300 kV using the Titan Krios microscope (Thermo Fisher Scientific), equipped with a K2 detector (Gatan), using SerialEM automated data collection software (v.3.8). Videos (32 frames, each 0.2 s, total dose 60 e^−^ Å^−2^) were recorded at a final pixel size of 0.82 Å with a defocus of between −1.2 and −2.0 μm. For PDF-2180 S trimer sample preparation, 3 μl of PDF-2180 S at 1 mg ml^−1^ was applied to a 2/2 C-flat grid (Protochips) that had been glow discharged for 30 s at 20 mA. The grids were plunge-frozen into liquid ethane using an FEI Mark IV Vitrobot with a 6.5–7.5 s blot time at 100% humidity and 20 °C. Data were acquired using the FEI Titan Krios transmission electron microscope operated at 300 kV and equipped with a Gatan K2 Summit direct detector and Gatan Quantum GIF energy filter, operated in zero-loss mode with a slit width of 20 eV. Automated data collection was carried out using Leginon (v.3.1)^56^ at a nominal magnification of ×130,000 with a super-resolution pixel size of 0.525 Å and a defocus range between −0.8 μm and −1.5 μm. The dose rate was adjusted to 8 counts per physical pixel per s, and each video was dose-fractionated in 50 frames of 200 ms.

### Image processing

For the NeoCoV RBD–Bat37ACE2 complex, a total of 4,234 micrographs were recorded. For the PDF-2018 RBD–Bat37ACE2 complex, a total of 3,298 micrographs were recorded. Both datasets were similarly processed. Raw data were processed using MotionCor2 (v.1.3.0). The raw data were aligned and averaged into motion-corrected summed images, after which, defocus values for each micrograph were determined using Gctf. Next, particles were picked and extracted for two-dimensional alignment. Well-defined partial particles were selected for initial model reconstruction in Relion^57^. The initial model was used as a reference for 3D classification. After refinement and post-processing, the overall resolution of the PDF-2018 RBD–Bat37ACE2 complex was up to 3.8 Å based on the gold-standard Fourier shell correlation (FSC; threshold = 0.143)^58^. For the NeoCoV RBD–Bat37ACE2 complex, the *C*_2_ symmetry was expanded before 3D refinement. Finally, the resolution of the NeoCoV RBD–Bat37ACE2 complex reached 3.5 Å. The quality of the local resolution was evaluated using ResMap (v.1.95)^59^. For the PDF-2180 spike trimer, a total of 1,746 micrographs were collected. Video frame alignment, estimation of the microscope contrast-transfer function parameters, particle picking and extraction were performed using Warp (v.1.0.9)^60^. Particles were extracted with a box size of 800 binned to 400 px^2^. Reference-free 2D classification was performed using Relion (v.3.0) to select well-defined particles images before 3D classification without symmetry applied using a MERS-CoV cryo-EM map^61^ as an initial model in Relion. 3D refinements and CTF refinement (to refine per-particle defocus values) were performed in Relion (v.3.0)^62^. Particle images were processed using the Bayesian polishing procedure implemented in Relion (v.3.0)^63^ before performing another round of 3D refinement and per-particle defocus refinement. Subsequently, 3D classification without alignment was performed using a mask focused on the N-terminal domain to improve its resolution, and the selected particles were processed for 3D refinement imposing *C*_3_ symmetry using non-uniform refinement in cryoSPARC (v.3.3.1)^64^, which yielded a final reconstruction of the PDF-2180 S at a resolution of 2.5 Å. Local-resolution estimation, filtering and sharpening were performed using CryoSPARC (v.3.3.1)^65^. Reported resolutions are based on the gold-standard FSC of 0.143 criterion, and FSC curves were corrected for the effects of soft masking by high-resolution noise substitution^66,67^.

### Model building and refinement

The NeoCoV RBD–Bat37ACE2 complex structures were manually built into the refined maps using Coot (v.0.9.4)^68^. The atomic models were further refined by positional and *B*-factor refinement in real space using Phenix (v.1.19)^59^. For building the PDF-2018 RBD–Bat37ACE2 complex model, the refinement NeoCoV RBD–Bat37ACE2 complex structures were manually docked into the refined maps using UCSF Chimera (v.1.15) and further corrected manually by real-space refinement in Coot. The atomic models were further refined by using Phenix (v.1.19) and Rosetta (v.1.2.5)^69,70^. For the PDF-2180 S model building, UCSF ChimeraX (v.1.1)^71^ and Coot were used to fit a MERS-CoV spike atomic model (PDB: 5W9J) into the PDF-2180 cryo-EM map. The model was subsequently manually rebuilt using Coot. *N*-linked glycans were hand built into the density where visible, and the models were rebuilt and refined using Rosetta (v.1.2.5)^69,70^. All of the models were validated and analysed using MolProbity^72^ and Privateer^73^. The figures were generated using ChimeraX. The datasets and refinement statistics are shown in Supplementary Table 1.

### Immunofluorescence assay

Expression levels of receptors were evaluated by immunofluorescence assay detecting the C-terminal 3×Flag tags. Cells expressing receptors were seeded in the 96-well plate (poly-lysine-pretreated plates for HEK293 cell lines) at a cell density of about 1–5 × 10^4^ cells per well and cultured for 24 h. Cells were fixed with 100% methanol at room temperature for 10 min and then incubated with a mouse monoclonal antibody (M2) targeting the Flag-tag (Sigma-Aldrich, F1804) diluted in 1% BSA/PBS at 37 °C for 1 h. After one wash with PBS, cells were incubated with 2 μg ml^−1^ of the Alexa Fluor 594-conjugated goat anti-mouse IgG (Thermo Fisher Scientific, A32742) diluted in 1% BSA/PBS at 37 °C for 1 h. The nuclei were stained with Hoechst 33342 (1:5,000 dilution in PBS). Images were captured with a fluorescence microscope (Mshot, MI52-N).

### Pseudotype virus production and titration

Coronavirus S pseudotyped viruses (CoV-PSVs) were generated according to a previously described protocol based on a replication-deficient VSV-based rhabdoviral pseudotyping system (VSV-dG)^74^. The VSV-G glycoprotein-deficient VSV co-expressing GFP and firefly luciferase (VSV-dG-GFP-fLuc) was rescued using a reverse genetics system in the laboratory and helper plasmids from Karafast. For CoV-PSV production, HEK293T or Vero E6 cells were transfected with the plasmids overexpressing the coronavirus spike proteins using the Lip2000 transfection reagent (Biosharp, BL623B). After 24–36 h, the transfected cells were transduced with VSV-dG-GFP-fLuc diluted in DMEM with 8 μg ml^−1^ polybrene for 4 h at 37 °C with a 50% tissue culture infectious dose (TCID_50_) of 1 × 10^6^ TCID_50_ per ml. After 2–5 h infection, virus inoculum was removed, and the cells were washed twice with DMEM or PBS before addition of DMEM supplemented with anti-VSV-G-antibody-containing supernatant (from IL-mouse hybridoma) with 10–50-fold dilution to minimize background from the parental viruses. CoV-PSV-containing supernatants were collected at 24 h after the medium change, clarified at 4,000 rpm for 5 min to remove cellular debris, aliquoted and frozen at −80 °C. The TCID_50_ of pseudotyped viruses was determined using a threefold-serial-dilution-based infection assay on HEK293T-bat40ACE2 cells for NeoCoV and PDF-2180 S pseudotypes, HEK293T-hDPP4 cells for MERS-CoV and HKU4 S pseudotypes, and BHK21-hACE2 cells for SARS-CoV-2 S pseudotypes. The TCID_50_ was calculated according to the Reed–Muench method^75,76^. Relative light unit values of ≥1,000 were considered to be positive. Viral titres (genome equivalents) of HKU5 and HKU31 without an ideal infection system were determined by quantitative PCR with reverse transcription using the HiScript II Q RT SuperMix (Vazyme, R223-01). RNA copies in the virus-containing supernatants were detected using primers for the VSV-N gene sequences (VSV-N-F, 5′-ACGGCGTACTTCCAGATGG-3′; VSV-N-R, 5′-CTCGGTTCAAGATCCAGGT-3′). For PDF-2180-, NeoCoV- and NeoCoV-T510F-neutralization assays, the S pseudotyped viruses were generated by transfecting the HEK293T cells using Lipofectamine 2000 (Life Technologies) according to the manufacturer’s instructions. After 5 h at 37 °C, DMEM supplemented with 20% FBS and 2% penicillin–streptomycin was added, and cells were incubated at 37 °C. The next day, cells were washed three times with DMEM and were transduced with VSVΔG-luc. After 2 h, virus inoculum was removed, and the cells were washed five times with DMEM before addition of DMEM supplemented with anti-VSV-G antibody (IL-mouse hybridoma supernatant diluted 1:25 (v/v)) to minimize parental background. After 18–24 h, the supernatants containing pseudotyped VSVs were collected, centrifuged at 2,000*g* for 5 min to remove cellular debris, filtered with a 0.45 µm membrane, concentrated ten times using a 30 kDa cut-off membrane (Amicon), aliquoted and frozen at −80 °C.

### Pseudotyped virus entry assay

Cells obtained by plasmid transfection or lentiviral transduction were trypsinized and incubated with different pseudotyped viruses (1 × 10^5^ TCID_50_ per 100 μl) in a 96-well plate (5 × 10^4^ per well) to allow attachment and entry simultaneously. For viruses without known susceptible cells, infections were performed using the same genome copies of a reference virus with a calculated TCID_50_ titre (commonly 1 × 10^5^ TCID_50_ per 100 μl). For TPCK-treated trypsin (Sigma-Aldrich, T8802) treatment, pseudotyped viruses in serum-free DMEM were incubated with 100 μg ml^−1^ TPCK-treated trypsin for 10 min at 25 °C. The reactions were stopped by adding 100 μg ml^−1^ soybean trypsin inhibitor (Sigma-Aldrich, T6414) in DMEM + 10% FBS. After 16–20 h, GFP images were acquired using a fluorescence microscope (Mshot, MI52-N), and intracellular luciferase activity was determined using the Bright-Glo Luciferase Assay Kit (Promega, E2620) and measured using the SpectraMax iD3 Multi-well Luminometer (Molecular Devices) or a GloMax 20/20 Luminometer (Promega).

### Pseudotyped virus neutralization assays

For viral RBD or soluble ACE2 neutralization assays, serial dilutions of proteins were prepared in DMEM. Pseudotyped viruses (1 × 10^5^ TCID_50_ per well) were mixed with 25 µl of each dilution and the mixtures were incubated for 30–45 min at 37 °C before addition to receptor-expressing cells seeded the day before at 2 × 10^4^ cells per well in a 96-well plate. After 16–20 h, the luciferase activity was measured in the same way as described for the pseudotype virus entry assay.

For pseudotyped virus neutralization with B6, SP6 and S2H14 IgGs, HEK293T cells were transfected with plasmids encoding for full-length hACE2 or Bat40 (*A. pallidus*) ACE2. In brief, HEK293T cells at 90% confluency were transfected using Lipofectamine 2000 (Life Technologies) and trypsinized at 5 h after transfection and seeded into 96-well plates at 50,000 cells per well overnight at 37 °C. For neutralizations, twofold serial dilutions of B6, S2P6 or S2H14 IgGs were prepared in DMEM. Then, 5 µl of the corresponding pseudotyped viruses was mixed with 20 µl of DMEM and 25 µl of each IgG dilution, and the mixtures were incubated for 45 min at 37 °C. Transfected HEK293T cells were washed three times with DMEM before adding 40 μl of the mixture containing virus and IgG. Then, 1 h later, 40 μl DMEM was added to the cells. After 17–20 h, 70 μl of One-Glo-EX substrate (Promega) was added to each well and incubated on a plate shaker in the dark. After 5–15 min incubation, the plates were read using the Biotek Neo2 plate reader. Measurements were taken in duplicate with biological replicates. Relative light units were plotted and normalized in Prism (GraphPad, v.8). Cells alone without pseudotyped virus was defined as 0% infection, and cells with virus only was defined as 100% infection.

### Western blot

Cells washed with PBS were lysed with 2% TritonX-100/PBS containing 1 mM freshly prepared PMSF (Beyotime, ST506) on ice for 10 min. Cell lysates were clarified by centrifugation at 12,000*g* at 4 °C for 5 min, mixed with 1:5 (v/v) 5× SDS-loading buffer, and incubated at 95 °C for 5 min. To detect the HA tag or VSV-M on pseudotyped viruses, 1 ml of virus-containing supernatant was incubated with 8% PEG at 4 °C overnight, precipitated by centrifugation at 7,000*g* for 10 min at 4 °C and resuspended with 50 μl 1×SDS loading buffer. After SDS–PAGE and PVDF membrane transfer, the blots were blocked with 5% milk in PBS containing 0.1% Tween-20 (PBST) or TBST (20 mM Tris-HCl pH 8.0, 150 mM NaCl) supplemented with 0.05% Tween-20 at room temperature for 1 h. Primary antibodies against Flag (Sigma-Aldrich, F1804), HA (BioLegend, 901515), VSV-M [23H12] (Kerafast, EB0011) and glyceraldehyde-3-phosphate dehydrogenase (GAPDH) (AntGene, ANT325) were added at a 1:10,000 dilution in PBST with 1% milk, or 1:250 dilution in TBST with 1% milk in the case of the stem-helix targeting monoclonal antibody B6, and incubated on a shaker at 4 °C. After three washes in PBST or TBST, the blots were incubated with horseradish peroxidase (HRP)-conjugated secondary antibody AffiniPure goat anti-mouse IgG (H+L) (Jackson Immuno Research, 115-035-003), AffiniPure goat anti-rabbit IgG (H+L) (Jackson Immuno Research, 111-035-003, 1:10,000 dilution) or Alexa Fluor 680-conjugated goat anti-human secondary antibody (1:50,000 dilution, Jackson ImmunoResearch, 109-625-098) for 1 hour. The blots were subsequently washed three times before visualization using the LI-COR Odyssey CLx or the Omni-ECL Femto Light Chemiluminescence Kit (EpiZyme, SQ201) and a ChemiDoc MP Imaging System (Bio-Rad). Information about antibodies is provided in Supplementary Table 3. Uncropped and unprocessed full scans of gel source data are provided in Supplementary Figs. 1 and 2.

### Coronavirus RBD–hFc live-cell binding assays

Recombinant coronavirus RBD–hFc proteins were diluted in DMEM at the indicated concentrations and then incubated with the cells for 1 h at 37 °C. After incubation, cells were washed once with DMEM and then incubated with 2 μg ml^−1^ of Alexa Fluor 488-conjugated goat anti-human IgG (Thermo Fisher Scientific; A11013) diluted in Hanks’ balanced salt solution (HBSS) with 1% BSA for 1 h at 37 °C. Cells were washed twice with PBS and incubated with Hoechst 33342 (1:5,000 dilution in HBSS) for nucleus staining. Images were captured using a fluorescence microscope (MI52-N). For flow cytometry analysis, cells were detached by 5 mM of EDTA/PBS and analysed using the CytoFLEX Flow Cytometer (Beckman). The dead cells, as indicated by SSC/FSC, were excluded by gating. A total of 10,000 events in a gated live-cell population were analysed for all of the samples. The RBD–hFc-bound cells were gated as indicated by the fluorescence intensity compared with HEK293T control cells without receptor expression. The flow cytometry data were analysed using FlowJo (v.10). The gating strategy for flow cytometry analysis (Fig. 2b) is exemplified in Supplementary Fig. 3.

### BLI binding assays

Protein binding affinities were determined using BLI assays performed on the Octet RED96 instrument (Molecular Devices). In brief, 20 μg ml^−1^ RBD–hFc recombinant proteins were loaded onto protein A (ProA) biosensors (ForteBio, 18–5010) for 30 s. The loaded biosensors were then dipped into the kinetic buffer (PBST) for 90 s to wash out unbound RBD–hFc proteins. Subsequently, biosensors were dipped into the kinetic buffer containing soluble ACE2 ectodomain proteins with concentrations ranging from 0 to 500 nM for 120 s to record association kinetic and then dipped into kinetics buffer for 300 s to record dissociation kinetics. Kinetic buffer without ACE2 was used to define the background. The corresponding affinities were calculated with the Octet Data Analysis software (v.12.2.0.20) using curve-fitting kinetic analysis or steady-state analysis with global fitting. *K*_D_,app values were reported because of the use of dimeric ACE2.

### ELISA

To evaluate binding between viral RBD and ACE2 in vitro, 96-well immuno-plates were coated with ACE2 ectodomains at the indicated concentrations in BSA/PBS (100 μl per well) overnight at 4 °C. After three washes with PBST, wells were blocked by 3% skimmed milk/PBS at 37 °C for 2 h. Next, different concentrations of RBD–hFc proteins (1–9 μg ml^−1^) diluted in 3% milk/PBST were added to the wells and incubated for 1 h at 37 °C. After extensive washes, wells were incubated with 1:2,000 diluted HRP-conjugated goat anti-human Fc antibody (Sigma-Aldrich, T8802) in 3% skimmed milk/PBS for 1 h at 37 °C. Finally, the substrate solution (Solarbio, PR1210) was added to the plates, and the absorbance at 450 nm after reaction termination was measured using the SpectraMax iD3 Multi-well Luminometer (Molecular Devices).

### Cell–cell fusion assays

A cell–cell fusion assay based on DSPs was conducted on HEK293T cells stably expressing different receptors^54^. Group A cells were transfected with spike and rLucN(1–155)-sfGFP1–7(1–157) expressing plasmids. Group B cells were transfected with spike and sfGFP8–11(158–231)-rLuc(156–311) expressing plasmids. Then, 12 h after transfection, cells from both groups were trypsinized and mixed into a 96-well plate at 8 × 10^4^ cells per well. Next, 24 h after transfection, cells were washed once with DMEM and then incubated with DMEM with or without 10–50 μg ml^−1^ TPCK-treated trypsin for 10 min at room temperature. Then, 6 h later, nuclei were stained with Hoechst 33342 (1:5,000 dilution in HBSS) for 30 min at 37 °C. Fluorescent images were subsequently captured using a fluorescence microscope (MI52-N; Mshot). For measurements of live-cell luciferase activity, 20 μM of EnduRen live-cell substrate (Promega, E6481) was added to the cells in DMEM and incubated for at least 1 h before detection using the Varioskan LUX Multi-well Luminometer (Thermo Fisher Scientific).

### Biosafety

The infection-related experiments were conducted in the State Key Laboratory of Virology, Wuhan University, strictly following the bio-safety regulations. Indeed, a VSV-based pseudotype virus system was used for all entry and neutralization assays, including new mutations in NeoCoV or PDF-2180 spikes that expand tropism for human cells. These pseudotyped viruses are non-replicating and non-pathogenic to humans and are commonly used by researchers to study the mechanisms of coronavirus entry and host range determination. The gain-of-function mutation, NeoCoV-T510F, was generated as we found that an Phe residue was present in PDF-2180 S at the equivalent position and its effect on binding affinity is consistent with our prediction models. We cautiously avoided extensively testing and showing the gain of function mutations on NeoCoV to expand its tropism on human cells.

### Statistical analysis

Most infection and live-cell binding-related experiments were repeated between 2 and 5 times with around 3–4 biological repeats. In vitro protein-binding-related experiments were conducted with 2–3 technical repeats. Similar results were obtained in all of the experiments, and representative data are shown. Data are presented as mean ± s.d. or mean ± s.e.m. as specified in the figure legends. Most statistical analyses were conducted using GraphPad Prism (v.8) using unpaired two-tailed Student’s *t*-tests, unless otherwise specified. *P* < 0.05 was considered to be significant; *P < 0.05, **P < 0.01, ***P < 0.005, ****P < 0.001.

### Reporting summary

Further information on research design is available in the Nature Portfolio Reporting Summary linked to this article.

## Online content

Any methods, additional references, Nature Portfolio reporting summaries, source data, extended data, supplementary information, acknowledgements, peer review information; details of author contributions and competing interests; and statements of data and code availability are available at 10.1038/s41586-022-05513-3.

## Supplementary information

Supplementary Information Supplementary Figs. 1–4 and Supplementary Tables 1–3.

Reporting Summary

Supplementary Table 4

## Data Availability

The cryo-EM maps have been deposited at the Electron Microscopy Data Bank under the following accession numbers: EMD-32686 (NeoCoV RBD–Bat37ACE2 complex), EMD-32693 (PDF-2180 RBD–Bat37ACE2 complex) and EMDB-26378 (PDF-2180 S trimer). Atomic models corresponding to EMD-32686, EMD-32693 and EMDB-26378 have been deposited at the PDB and are available under the following accession numbers: 7WPO, 7WPZ and 7U6R, respectively. The accession numbers (NCBI Genbank or GISAID), protein sequences or species information of receptor, viral, antibody and reporter genes are provided in the Methods and Supplementary Table 4. All other data supporting the findings of this study are available with the Article and the Supplementary Information. Source data are provided with this paper.

## Source data

Source Data Fig. 1
Source Data Fig. 2
Source Data Fig. 3
Source Data Fig. 4
Source Data Fig. 5
Source Data Extended Data Fig. 1
Source Data Extended Data Fig. 3
Source Data Extended Data Fig. 5
Source Data Extended Data Fig. 8
Source Data Extended Data Fig. 10
