# Supplementary material for: Close relatives of MERS-CoV in bats use ACE2 as their functional receptors
Source: Nature. 2022 Dec 7;612(7941):748–57. doi: 10.1038/s41586-022-05513-3 (PMC9734910; doi:10.1038/s41586-022-05513-3)
Supplement: Supplementary file 1 — Supplementary Figs. 1–4 and Supplementary Tables 1–3. [file 41586_2022_5513_MOESM1_ESM.pdf]

nature portfolio

https://doi.org/10.1038/s41586-022-05513-3

Supplementary information

Close relatives of MERS-CoV in bats use ACE2 as their functional receptors

In the format provided by the authors and unedited

Supplementary information

Close relatives of MERS-CoV in bats use ACE2 as their functional receptors

Qing Xiong[1,%], Lei Cao[2,%], Chengbao Ma[1,%], M. Alejandra Tortorici[3,%], Chen Liu[1], Junyu Si[1], Peng Liu[1], Mengxue Gu[1], Alexandra C. Walls[3,4], Chunli Wang[1], Lulu Shi[1], Fei Tong[1], Meiling Huang[1], Jing Li[1], Chufeng Zhao[1], Chao Shen[1], Yu Chen[1], Huabin Zhao[5], Ke Lan[1], Davide Corti[6], David Veesler[3,4,*], Xiangxi Wang[2,7,*], Huan Yan[1,*]

[1] State Key Laboratory of Virology, Institute for Vaccine Research and Modern Virology Research Center, College of Life Sciences, TaiKang Center for Life and Medical Sciences, Wuhan University, Wuhan, Hubei, 430072, China.

[2] CAS Key Laboratory of Infection and Immunity, National Laboratory of Macromolecules, Institute of Biophysics, Chinese Academy of Sciences, Beijing, 100101, China.

[3] Department of Biochemistry, University of Washington, Seattle, WA 98195, USA.

[4] Howard Hughes Medical Institute, Seattle, WA 98195, USA.

[5] Department of Ecology, Tibetan Centre for Ecology and Conservation at WHU-TU, Hubei Key Laboratory of Cell Homeostasis, College of Life Sciences, Wuhan University, Wuhan 430072, China

[6] Humabs BioMed SA, subsidiary of Vir Biotechnology, 6500 Bellinzona, Switzerland

[7] University of Chinese Academy of Sciences, Beijing, 100049, China.

[%] These authors contributed equally to this work.

[*] Correspondence: huanyan@whu.edu.cn, xiangxi@ibp.ac.cn, dveesler@uw.edu

Table of content:

SI Figure 1. Uncropped immunoblots from Main Figures.

SI Figure 2. Uncropped immunoblots from Extended Data Figures.

SI Figure 3. Gating strategies for flow cytometry analysis of RBD-hFc binding to ACE2 expressing HEK293T cells in Figure 2b.

SI Figure 4. Original and fitted curves of BLI data.

SI Table 1. Cryo-EM data collection, refinement and validation statistics.

SI Table 2. Residues of NeoCoV RBD and PDF-2180 RBD interacting with Bat37ACE2 at the binding interface (using a cutoff distance of 4.5 Å).

SI Table 3. Information on antibodies used in this study.

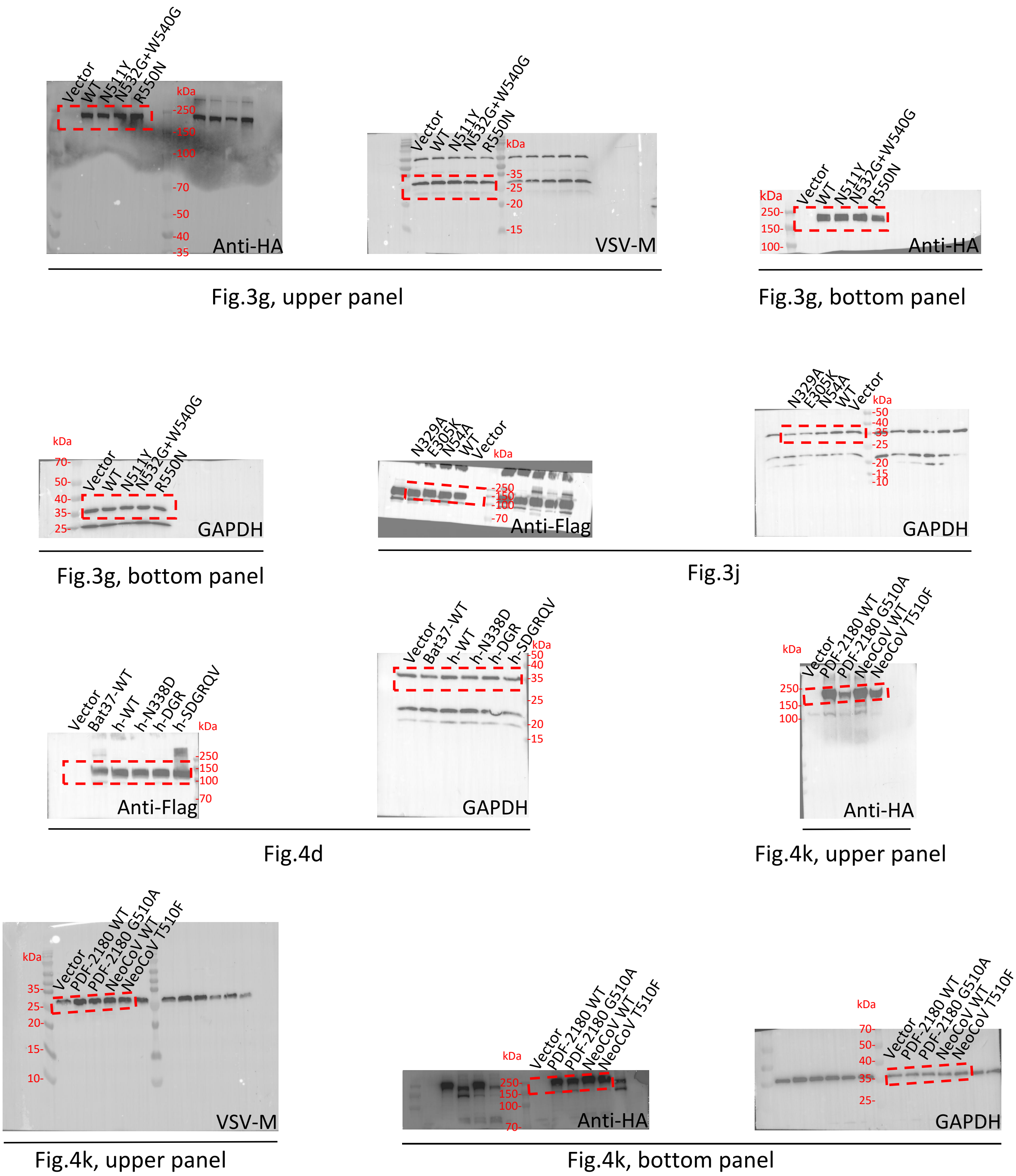

SI Figure 1. Uncropped immunoblots from Main Figures. Uncropped and unprocessed scans of the protein blots used in Main Figures. Molecular weight markers, detecting antibodies, and the corresponding Figures were indicated.

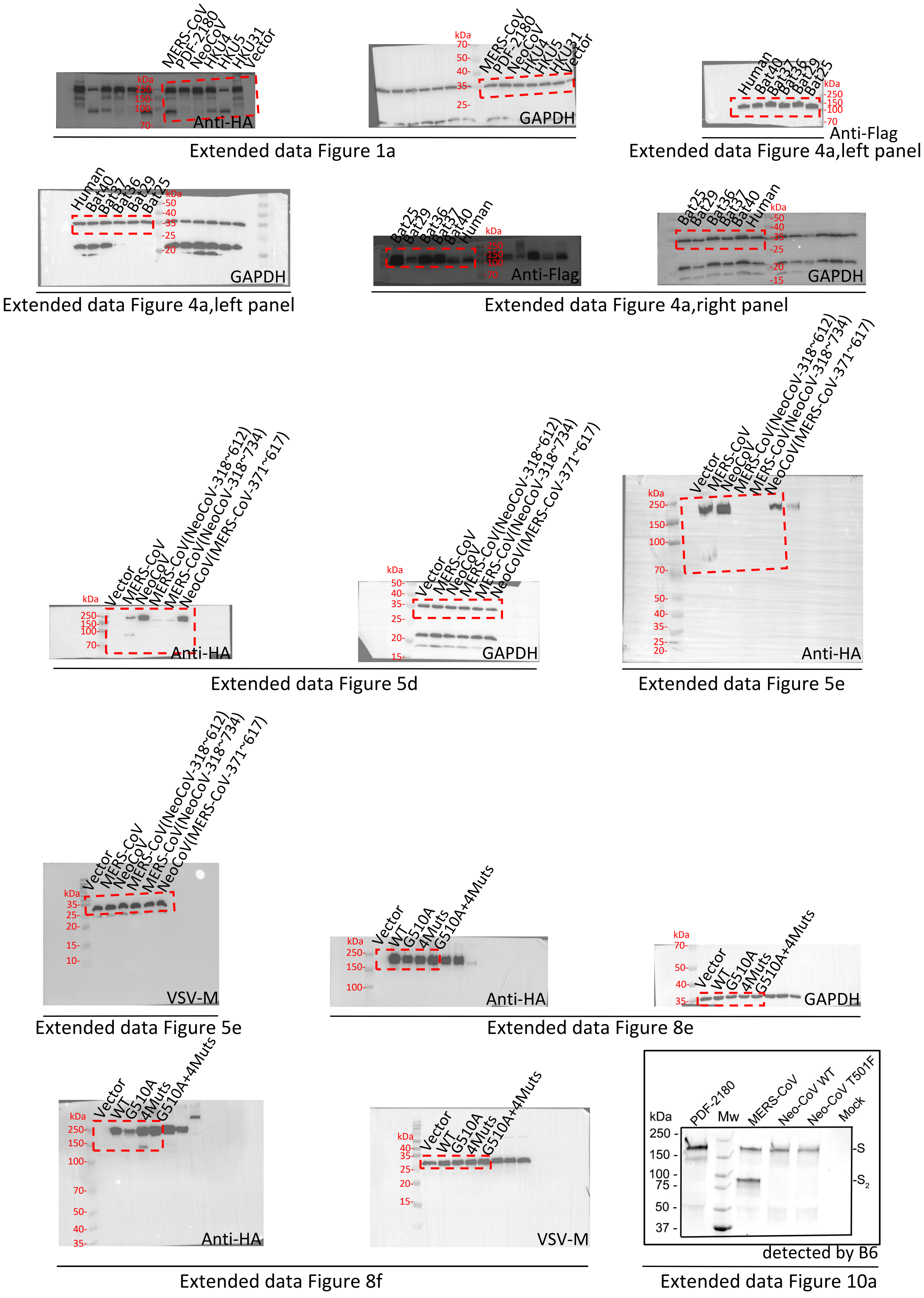

SI Figure 2. Uncropped immunoblots from Extended Data Figures. Uncropped and unprocessed scans of the protein blots used in Extended data Figures. Molecular weight markers, detecting antibodies, and the corresponding Figures were indicated.

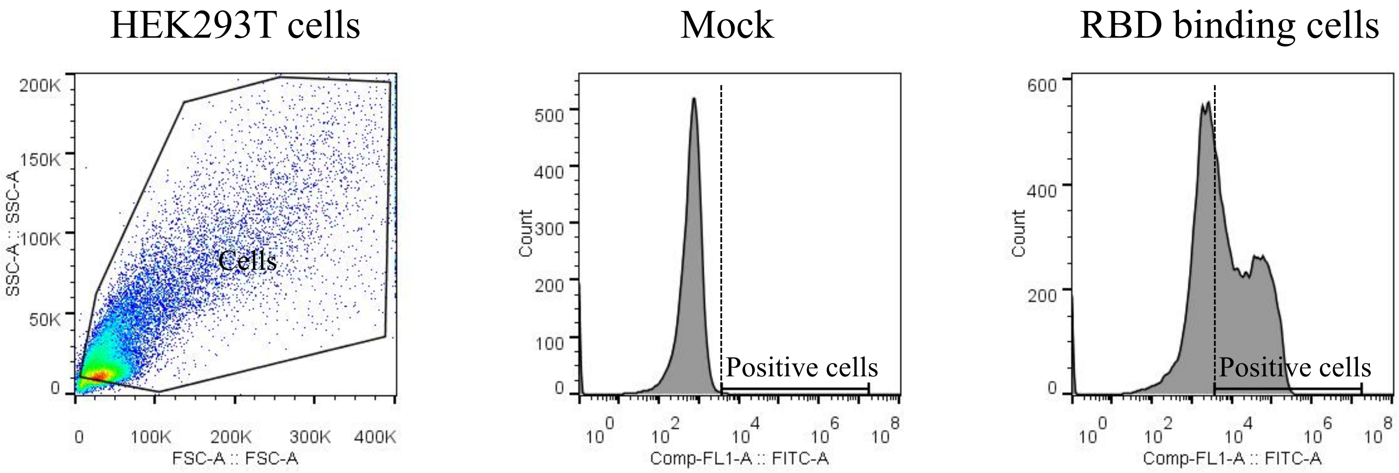

SI Figure 3. Gating strategies for flow cytometry analysis of RBD-hFc binding to ACE2 expressing HEK293T cells in Figure 2b. Representative gating to exclude cell debris and dead cells (FSC-A/SSC-A) and to select RBD binding positive cells (FITC-A) based on the threshold set based on the histogram of mock control (HEK293T transfected with vector plasmids only).

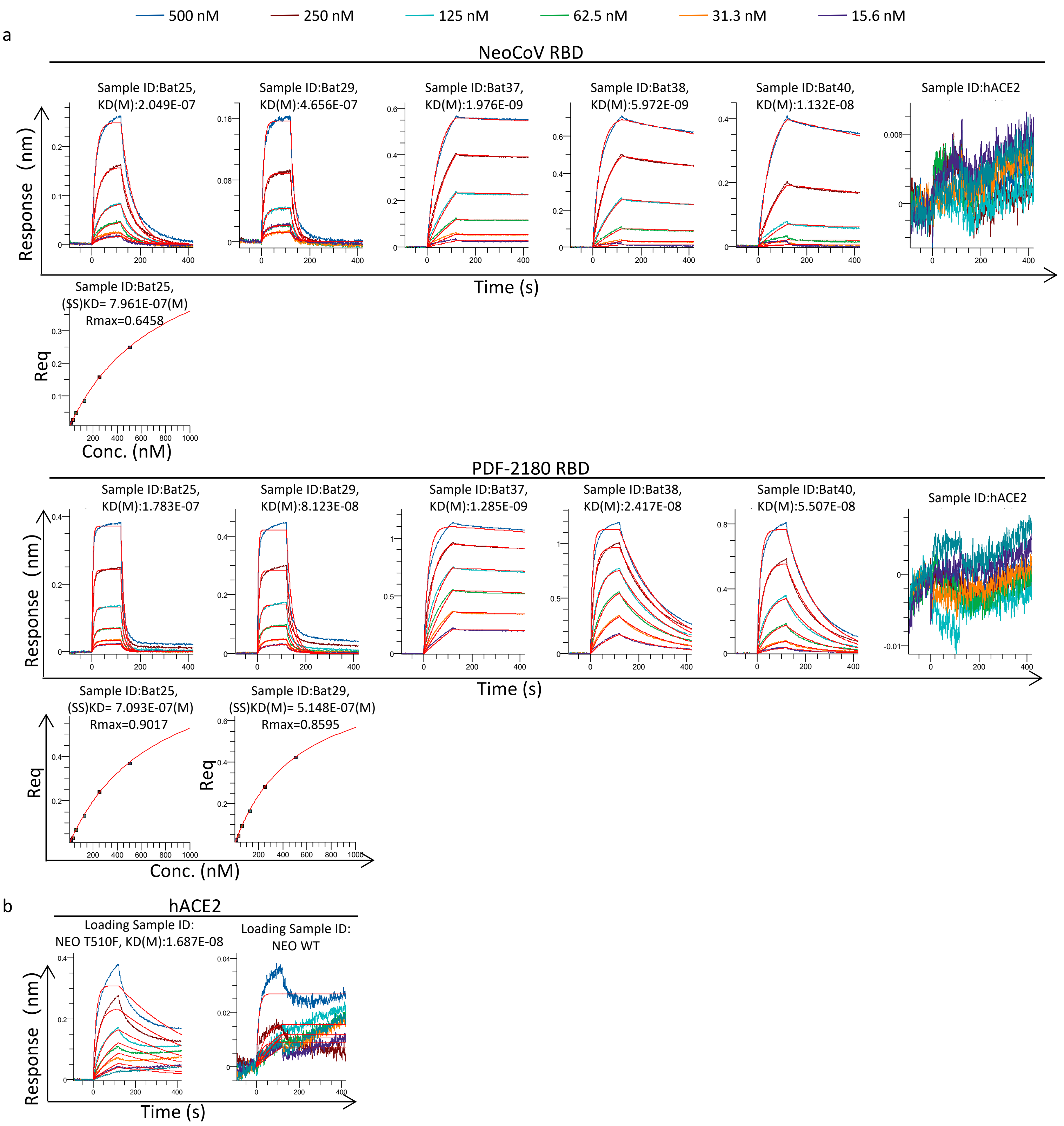

SI Figure 4. Original and fitted curves of BLI data. a, Binding kinetics of interaction between NeoCoV or PDF-2180 RBD and the indicated ACE2 orthologs. The fitted curves are also presented in **Fig. 2c**. Steady-state affinity determination was applied to Bat25ACE2 and Bat29ACE2, and steady-state graphs are shown below. **b**, Binding kinetics of interaction between NeoCoV WT or NeoCoV T510F RBD and hACE2. The fitted curves were also presented in **Extended data Fig. 8b**.

SI Table 1. Cryo-EM data collection, refinement and validation statistics

	Neo-CoV RBD-Bat37 ACE2 complex (EMDB-32686) (PDB 7WPO)	PDF-2018 RBD-Bat37 ACE2 complex (EMDB-32693) (PDB 7WPZ)	PDF-2018 Spike (EMDB-26378) (PDB 7U6R)
**Data collection and processing**			
Magnification	130,000	130,000	130,000
Voltage (kV)	300	300	300
Electron exposure (e–/Å^2)	60	60	70
Defocus range (μm)	-1.2 ~-1.8	-1.2 ~-1.8	-0.8~-1.5
Pixel size (Å)	1.04	1.04	0.525
Symmetry imposed	C1	C1	C3
Initial particle images (no.)	654,214	131,254	162,727
Final particle images (no.)	162,615	80,090	47,094
Map resolution (Å)	3.5	3.8	2.5
FSC threshold			
Map resolution range (Å)	3.5-60	3.8-60	2.5-60
**Refinement**			
Initial model used (PDB code)	none	none	5W9J
Model resolution (Å)	3.7	4.2	2.8
FSC threshold	0.5	0.5	0.5
Map sharpening *B* factor (Å^2)	-200	-130	-200
Model composition			
Non-hydrogen atoms	6,829	5756	51,819
Protein residues	867	787	3,303
Ligands	17	15	51
B factors (Å^2)			
Protein	42.2	64.88	20.74
Ligand	46.2	80.02	59.29
R.m.s. deviations			
Bond lengths (Å)	0.013	0.004	0.016
Bond angles (°)	1.56	0.597	1.573
Validation			
MolProbity score	1.4	1.35	0.64
Clashscore	5.14	4.42	0.37
Poor rotamers (%)	1.06	0.25	0.1
Ramachandran plot			
Favored (%)	97.32	97.3	97.95
Allowed (%)	2.56	2.7	1.95
Disallowed (%)	0.12	0.00	0.09

SI Table 2. Residues of NeoCoV RBD and PDF-2180 RBD interacting with Bat37ACE2 at the binding interface (using a cutoff distance of 4.5 Å)

Bat37ACE2	NeoCoV RBD	Bat37ACE2	PDF-2180 RBD
Residues	Residues	Residues	Residues
E305	T510	A304	F511
	K512	E305	F511
W328	A509		K513
N329	G508	F308	F511
	A509	W328	G510
	G546		F511
	P548	N329	G509
S331	A509		G510
M332	A509		G547
L333	A509		P549
	T510	N330	N544
T334	A509		G547
	T510		P549
	N511	S331	G510
E335	N511	M332	G510
P336	N511	L333	G510
D338	N504		F511
	N506	T334	F511
	L539		N512
R340	N511	E335	N512
	R550	P336	N512
		D338	N505
			N507
			L540
		R340	N512
			R551

SI Table 3. Information on antibodies used in this study

Reagent type	Designation	Host	Source (clone/catalog number/PMID and dilution/concentration for antibodies)
Antibody, WB, IFA	Anti-Flag	Mouse	Sigma, F1804/clone M2 (WB, 1:10,000; IFA, 1:1,000)
Antibody, WB	Anti-HA.11 epitope tag antibody	Mouse	Biolegend, 901515/clone 16B12 (1:10,000)
Antibody, WB	Anti-VSV-M	Mouse	Kerafast, EB0011/clone 23H12 (1:10,000)
Antibody, WB	GAPDH Polyclonal Antibody	Rabbit	AntGene, ANT325 (1:10,000)
Antibody, WB	Stem-helix- B6 monoclonal antibody	Mouse	Clone: B6; PMID: 33981021 (1:250)
Secondary antibody, IFA	Alexa Fluor 594-conjugated goat anti-mouse IgG	Goat	Thermo Fisher Scientific, A32742 (1:1,000)
Secondary antibody, IFA	Alexa Fluor 488-conjugated goat anti-human IgG	Goat	Thermo Fisher Scientific, A11013 (1:1,000)
Secondary antibody, WB	AffiniPure Goat Anti-Mouse IgG (H+L)	Goat	Jackson ImmunoResearch, 115-035-003 (1:10,000)
Secondary antibody, WB	AffiniPure Goat Anti-Rabbit IgG (H+L)	Goat	Jackson ImmunoResearch, 111-035-003 (1:10,000)
Secondary antibody, WB	Alexa Fluor 680-conjugated goat anti-human secondary antibody	Goat	Jackson ImmunoResearch, 109-625-098 (1:50,000)
Neutralizing antibody	H11B11	Human	Clone: H11B11; PMID: 34404805
Neutralizing antibody	Anti-VSVG	Mouse	ATCC: I1-Hybridoma (CRL-2700)
Neutralizing antibody	S2P6	Human	Clone: S2P6; PMID: 34344823
Neutralizing antibody	B6	Mouse	Clone: B6; PMID: 33981021
Neutralizing antibody	S2H14	Human	Clone: S2H14; PMID: 32991844
