# Supplementary material for: Close relatives of MERS-CoV in bats use ACE2 as their functional receptors
Source: Nature. 2022 Dec 7;612(7941):748–57. doi: 10.1038/s41586-022-05513-3 (PMC9734910; doi:10.1038/s41586-022-05513-3)
Supplement: Supplementary file 2 — Reporting Summary [file 41586_2022_5513_MOESM2_ESM.pdf]

nature portfolio

Corresponding author(s): Huan Yan

Last updated by author(s): Sep 29, 2022

Reporting Summary

Nature Portfolio wishes to improve the reproducibility of the work that we publish. This form provides structure for consistency and transparency in reporting. For further information on Nature Portfolio policies, see our Editorial Policies and the Editorial Policy Checklist.

Statistics

For all statistical analyses, confirm that the following items are present in the figure legend, table legend, main text, or Methods section.

n/a	Confirmed	
☐	☒	The exact sample size (*n*) for each experimental group/condition, given as a discrete number and unit of measurement
☐	☒	A statement on whether measurements were taken from distinct samples or whether the same sample was measured repeatedly
☐	☒	The statistical test(s) used AND whether they are one- or two-sided<br>*Only common tests should be described solely by name; describe more complex techniques in the Methods section.*
☒	☐	A description of all covariates tested
☒	☐	A description of any assumptions or corrections, such as tests of normality and adjustment for multiple comparisons
☐	☒	A full description of the statistical parameters including central tendency (e.g. means) or other basic estimates (e.g. regression coefficient) AND variation (e.g. standard deviation) or associated estimates of uncertainty (e.g. confidence intervals)
☐	☒	For null hypothesis testing, the test statistic (e.g. *F*, *t*, *r*) with confidence intervals, effect sizes, degrees of freedom and *P* value noted<br>*Give P values as exact values whenever suitable.*
☒	☐	For Bayesian analysis, information on the choice of priors and Markov chain Monte Carlo settings
☒	☐	For hierarchical and complex designs, identification of the appropriate level for tests and full reporting of outcomes
☒	☐	Estimates of effect sizes (e.g. Cohen's *d*, Pearson's *r*), indicating how they were calculated

Our web collection on statistics for biologists contains articles on many of the points above.

Software and code

Policy information about availability of computer code

Data collection	Western blot data are collected by ChemiDoc MP (Bio-Rad). Spectra MaxiD3 multi-well Luminometer (Molecular Devices, United States) , GloMax® 20/20 Luminometer (Promega, United States) , Varioskan LUX Multi-well Luminometer (Thermo Fisher) ,or Biotek Neo2 plate reader for measuring the luciferase activity. CytoFLEX Flow Cytometer (Beckman Coulter, United States) for generating the flow cytometry data. For cryoEM analysis, micrographs were collected at 300 kV using a Titan Krios microscope (Thermo Fisher), equipped with a K2 or K3/GIF detector (Gatan, Pleasanton, CA), using SerialEM (version 3.8) or Leginon(version 3.1) automated data collection software. BLI assays were performed on an Octet RED96 instrument (Molecular Devices, United States). Fluorescent images were captured with a fluorescence microscope (Mshot, MI52-N).
Data analysis	GraphPad Prism(version 8) was used for all statistical analyses. FlowJo(version 10) was used for analyzing flow cytometry data. Protein sequence alignment and phylogenetic analysis were performed using the MEGA-X software (version 10.1.8) and ClustalW (https://www.genome.jp/tools-bin/clustalw). The nucleotide similarity of coronaviruses was analyzed by SimPlot software (version 3.5.1). The raw data of the Cryo-EM were processed by MotionCor2 (version 1.3.0); the defocus value for each micrograph was determined using Gctf; the preprocessing was done using Warp (version 1.0.9). The well-defined partial particles were selected for initial model reconstruction in Relion (version 3.0) and final reconstructions used either Relion (version 3.0) or cryoSPARC (version 3.3.1). Local resolution was evaluated using ResMap(version 1.95) or cryoSPARC(version 3.3.1). The protein complex structures were manually built into the refined maps in Coot (version 0.9.4), UCSF Chimera (version 1.15) or UCSF ChimeraX (version 1.1). The atomic models were further refined by positional and B-factor refinement in real space using Phenix (version 1.19) or Rosetta (version 1.2.5). Validation of the final model was performed with Molprobity and Privateer. Molecular dynamics (MD) prediction of the effect of residue mutations on protein-protein interactions was conducted by mCSM-PPI2 (http://biosig.unimelb.edu.au/mcsm_ppi2/). The BLI data were analyzed by Octet Data Analysis software 12.2.0.20.

For manuscripts utilizing custom algorithms or software that are central to the research but not yet described in published literature, software must be made available to editors and reviewers. We strongly encourage code deposition in a community repository (e.g. GitHub). See the Nature Portfolio guidelines for submitting code & software for further information.

Data

Policy information about availability of data

All manuscripts must include a data availability statement. This statement should provide the following information, where applicable:

- Accession codes, unique identifiers, or web links for publicly available datasets
- A description of any restrictions on data availability
- For clinical datasets or third party data, please ensure that the statement adheres to our policy

The cryo-EM maps have been deposited at the Electron Microscopy Data Bank (www.ebi.ac.uk/emdb) and are available under accession numbers: EMD-32686 (NeoCoV RBD-Bat37ACE2 complex), EMD-32693 (PDF-2180 RBD-Bat37ACE2 complex), and EMDB-26378 (PDF-2180 S trimer). Atomic models corresponding to EMD-32686, EMD-32693 and EMDB-26378 have been deposited in the Protein Data Bank (www.rcsb.org) and are available under accession numbers PDB 7WPO, PDB 7WPZ, PDB 7U6R, respectively. The accession numbers (NCBI Genbank or GISAID), protein sequences, species information of receptor, viral, antibody, and reporter genes were accessible in the method section and Supplementary Table 4. The authors declare that all other data supporting the findings of this study are available with the paper and its supplementary information files.

Field-specific reporting

Please select the one below that is the best fit for your research. If you are not sure, read the appropriate sections before making your selection.

☒ Life sciences ☐ Behavioural & social sciences ☐ Ecological, evolutionary & environmental sciences

For a reference copy of the document with all sections, see nature.com/documents/nr-reporting-summary-flat.pdf

Life sciences study design

All studies must disclose on these points even when the disclosure is negative.

Sample size

In this study, we analyzed 46 bat ACE2 orthologs for their ability to support viral binding and entry. This size is determined by the size of an established stable cell library in a previous study (Yan et al, 2021, Nature Ecol. &Evol. PMID: 33649547). The size of the library is restricted by the number of bat species with available bat ACE2 sequences, and this sampling covers representative species from 11 bat families, hence providing a broad picture of bat phylogeny.

We tested six bat DPP4 orthologs to exclude their potential role in NeoCoV and PDF-2180 entry. These six species are selected as they are vesper bats (close relatives of NeoCoV and PDF-2180 hosts) and their ACE2 alleles promote robust NeoCoV and PDF-2180 spike pseudotype virus entry. DPP4 orthologs from none of these species showed any activity in supporting NeoCoV/PDF-2180 spike-mediated binding and entry. Therefore, we think the sample size is sufficient to exclude a potential role of DPP4 in supporting the entry of these viruses.

In serum and nanobody neutralization assays, we used ten SARS-CoV-2 vaccinated sera and ten MERS-RBD specific nanobodies to demonstrate the poor neutralizing activity of these antibodies against NeoCoV and PDF-2180. The sample size of the sera is restricted by the availability of samples with high anti-SARS-CoV-2 neutralizing activity. The sample size of the nanobodies is determined based on the number of sera samples, as we wanted the two related assays to be done in parallel. Theoretically, these antibodies were anticipated to be deficient in recognizing NeoCoV and PDF-2180, considering their distinct spike, especially RBD, structures compared with SARS-CoV-2. Thus, we think the size of ten is reasonable to support our conclusions.

Data exclusions

There is no data excluded from the analysis.

Replication

Experiments were repeated 2~5 times with 2-4 biological replicates, each yielding similar results. Most of the assays were conducted three times with biological triplicates, which is sufficient to show the reproducibility of our experimental data. Overall, our results are highly reproducible as most of them are well-established cell-based in vitro assays with strict controls. Some experiments were conducted twice as these data exhibited clear-cut differences between groups, with most of the P values <0.001 (****). All findings described in this study could be replicated or reproduced.

Randomization

We used a previously described bat ACE2-expressing cell library in this study. As we are the first to show the potential role of ACE2 in the spike-mediated entry of NeoCoV and PDF-2180, we attempt to include as many bat ACE2 sequences as possible. Therefore ACE2 species randomization is not relevant to our study. As for the serum neutralization assay, we aimed to explore whether SARS-CoV-2 specific antibodies can protect the vaccinated individuals from NeoCoV and PDF-2180 infection. Our rationale is to allocate as many as well-vaccinated individuals with confirmed sera neutralization of SARS-CoV-2 entry with limited human sera samples. The effect of covariates on sera neutralization is beyond the scope of this study. Thus, randomization is not performed in our experiment design.

Blinding

No grouping is applied in this study. Therefore blinding is not applicable here.

Reporting for specific materials, systems and methods

We require information from authors about some types of materials, experimental systems and methods used in many studies. Here, indicate whether each material, system or method listed is relevant to your study. If you are not sure if a list item applies to your research, read the appropriate section before selecting a response.

Materials & experimental systems

n/a	Involved in the study
☐	☒ Antibodies
☐	☒ Eukaryotic cell lines
☒	☐ Palaeontology and archaeology
☒	☐ Animals and other organisms
☐	☒ Human research participants
☒	☐ Clinical data
☐	☒ Dual use research of concern

Methods

n/a	Involved in the study
☒	☐ ChIP-seq
☐	☒ Flow cytometry
☒	☐ MRI-based neuroimaging

Antibodies

Antibodies used	Primary antibodies:<br>Anti-Flag Mouse Sigma, F1804/clone M2 (WB, 1:10,000; IFA,1:1,000); Anti-HA.11 epitope tag antibody Mouse Biolegend, 901515/ clone 16B12 (WB,1:10,000); Anti-VSV-M Mouse Kerafast, EB0011/clone 23H12 (WB,1:10,000); GAPDH Polyclonal Antibody Rabbit AntGene, ANT325 (WB,1:10,000); Stem-helix-B6 monoclonal antibody, Mouse Clone: B6; PMID: 33981021 (WB1:250⍰<br>Secondary antibodies:<br>Alexa Fluor 594-conjugated goat anti-mouse IgG, Goat, Thermo Fisher Scientific, A32742 (IFA, 1:1,000); Alexa Fluor 488-conjugated goat anti-human IgG, Goat Thermo Fisher Scientific, A11013 (IFA,1:1,000); AffiniPure Goat Anti-Mouse IgG (H+L) Goat, Jackson Labs, 115-035-003 (WB,1:10,000⍰; AffiniPure Goat Anti-Rabbit IgG⍰H+L⍰Goat, Jackson Labs, 111-035-003 (WB,1:10,000); Alexa Fluor 680-conjugated goat anti-human secondary antibody, Goat, Jackson ImmunoResearch, 109-625-098,⍰WB, 1:50,000⍰<br><br>Neutralizing antibodies:<br>H11B11, Human Clone: H11B11; PMID: 34404805; Anti-VSVG, Mouse ATCC: I1-Hybridoma (CRL-2700) cell line; S2P6, Human Clone: S2P6; PMID: 34344823; B6, Mouse Clone: B6; PMID: 33981021; S2H14, Human Clone: S2H14; PMID: 32991844<br><br>A summary of antibodies used in this study is now provided in the supplementary information.
Validation	H11B11 (PMID: 34404805), B6 (PMID: 33981021), S2P6 (PMID: 34344823) and S2H14 (PMID: 32991844) were sequence-verified in the indicated papers. All other antibodies are validated by the vendors.

Eukaryotic cell lines

Policy information about cell lines

Cell line source(s)	HEK293T (CRL-3216), Vero E6 cells (CRL-1586), A549 (CCL-185), BHK-21 (CCL-10), Caco-2 (HTB-37) ,Tb 1 Lu (CCL-88), and I1-Hybridoma (CRL-2700) were purchased from ATCC. Huh-7 (SCSP-526) cells were purchased from the Cell Bank of Type Culture Collection, Chinese Academy of Sciences. HEK Expi 293F cells (A14527) were purchased from Thermo Fisher.
Authentication	Not authenticated.
Mycoplasma contamination	Cell lines used in this study were not tested for mycoplasma contamination.
Commonly misidentified lines<br>(See ICLAC register)	No commonly misidentified line was used.

Human research participants

Policy information about studies involving human research participants

Population characteristics	All the vaccinated sera were collected from volunteers about 21 days post the third dose of the WHO-approved inactivated SARS-CoV-2 vaccine (CorovaVac, Sinovac, China). Median age of volunteers was 37 years. 44% of participants were males, and 56% were females.
Recruitment	All of the volunteers were recruited by Sinovac, Inc. None of the participants had a history of prior SARS-CoV-2 infection, and none reported serious adverse events after vaccination. All volunteers were provided informed written consent forms, and the whole study was conducted following the requirements of Good Clinical Practice of China.
Ethics oversight	The procedures for human participants were approved by the Ethics Committee (seal) of Beijing Youan Hospital, Capital Medical University, with an approval number of LL-2021-042-K.

Note that full information on the approval of the study protocol must also be provided in the manuscript.

Dual use research of concern

Policy information about dual use research of concern

Hazards

Could the accidental, deliberate or reckless misuse of agents or technologies generated in the work, or the application of information presented in the manuscript, pose a threat to:

No	Yes	
☒	☐	Public health
☒	☐	National security
☒	☐	Crops and/or livestock
☒	☐	Ecosystems
☒	☐	Any other significant area

Experiments of concern

Does the work involve any of these experiments of concern:

No	Yes	
☒	☐	Demonstrate how to render a vaccine ineffective
☒	☐	Confer resistance to therapeutically useful antibiotics or antiviral agents
☒	☐	Enhance the virulence of a pathogen or render a nonpathogen virulent
☒	☐	Increase transmissibility of a pathogen
☐	☒	Alter the host range of a pathogen
☒	☐	Enable evasion of diagnostic/detection modalities
☒	☐	Enable the weaponization of a biological agent or toxin
☒	☐	Any other potentially harmful combination of experiments and agents

Precautions and benefits

Biosecurity precautions	We used a VSV-based pseudovirus system with only single round entry ability for all entry and neutralization assays, including creating mutations in the NeoCoV and PDF-2180 spikes that expand tropism to human cells. These pseudoviruses are non-replicating and nonpathogenic to humans and are widely used by researchers to study the mechanism of host range determination. To investigate the host range determination and human emergence risk of these viruses, the NeoCoV T510F mutation was investigated and we found an F amino acid at the corresponding site of the PDF-2180 spike, which is also consistent with our predicted model of increased hACE2 binding. We similarly investigated the PDF-2180 G510A spike mutation. None of these pseudovirus pose a threat to public health. This study did NOT produce or engineer authentic NeoCoV or PDF-2180 viruses by reverse genetics, considering the potential biosafety issues. We do not encourage efforts to rescue the replication-competent NeoCoV authentics viruses with mutations with human emergence potential such as T510F. We suggest these experiments be strictly limited to the Biosafety Level III or IV laboratory, if necessary.
Biosecurity oversight	Only a pseudovirus system was used for all spike-mediated entry experiments, which are considered safe and supervised by the State Key Laboratory of Virology, Wuhan University or the University of Washington, Seattle.
Benefits	Our study underscores the existence of a group of potentially zoonotic viruses. Whether these viruses will pose a risk for humans deserves further study. Identifying a potential mutation that could allow a virus to infect human cells is critical for assessing the risks of the host jumping through viral evolution. Our study highlighted the importance of surveillance and research of these zoonotic viruses to prepare for possible outbreaks in the future, which is beneficial to public health in the long run (for pandemic preparedness).
Communication benefits	Our study underscores that there is more than a few human coronaviruses that we should pay attention to. It is also beneficial for people to understand that "to identify putatively high-risk viruses before they emerge" is a crucial scientific task. The awareness of the risk is helpful for people to respect nature and keep their distance from wild animals, which in turn reduces the risk of spillover events.

Flow Cytometry

Plots

Confirm that:

☒ The axis labels state the marker and fluorochrome used (e.g. CD4-FITC).

☒ The axis scales are clearly visible. Include numbers along axes only for bottom left plot of group (a 'group' is an analysis of identical markers).

☒ All plots are contour plots with outliers or pseudocolor plots.

☒ A numerical value for number of cells or percentage (with statistics) is provided.

Methodology

Sample preparation	The HEK293T cells stably overexpressing different ACE2 orthologs were incubated with RBDs and antibodies in two

Sample preparation	consecutive staining steps. Cells were firstly incubated with virus RBD-hFc (5-10 μg/ml) in DMEM with 2% FBS at 37°C for one hour. Next, cells were incubated with an Alexa Fluor 488 conjugated Goat anti-Human IgG (A11013, Thermo Fisher Scientific, United States) at 2 μg/ml for an additional 30min at 37°C. The stained cells were detached by 5mM EDTA/PBS before passing through a 70μm cell mesh, and then analyzed with a CytoFLEX Flow Cytometer (Beckman Coulter, United States).
Instrument	CytoFLEX Flow Cytometer (Beckman Coulter, United States).
Software	FlowJo version 10
Cell population abundance	10,000 events in the gated area were analyzed for all samples. Only HEK-293T cells were used in the analysis without sorting.
Gating strategy	Representative gating to exclude cell debris and dead cells (FSC-A/SSC-A) and to select RBD binding positive cells (FITC-A) based on the threshold set based on the histogram of mock control (HEK293T transfected with vector plasmids only).<br>A figure describing the gating strategy of main Figure 2b is now included in supplementary Figure 3.

☒ Tick this box to confirm that a figure exemplifying the gating strategy is provided in the Supplementary Information.
